# Crystal Structures of *Wolbachia* CidA and CidB Reveal Determinants of Bacteria-induced Cytoplasmic Incompatibility and Rescue

**DOI:** 10.1038/s41467-022-29273-w

**Published:** 2022-03-25

**Authors:** Haofeng Wang, Yunjie Xiao, Xia Chen, Mengwen Zhang, Guangxin Sun, Feng Wang, Lin Wang, Hanxiao Zhang, Xiaoyu Zhang, Xin Yang, Wenling Li, Yi Wei, Deqiang Yao, Bing Zhang, Jun Li, Wen Cui, Fenghua Wang, Cheng Chen, Wei Shen, Dan Su, Fang Bai, Jinhai Huang, Sheng Ye, Lei Zhang, Xiaoyun Ji, Wei Wang, Zefang Wang, Mark Hochstrasser, Haitao Yang

**Affiliations:** 1 School of Life Sciences, Tianjin University, Tianjin, China; 2 Shanghai Institute for Advanced Immunochemical Studies and School of Life Science and Technology, ShanghaiTech University, Shanghai, China; 3 Shanghai Clinical Research and Trial Center, Shanghai, China; 4 Institute of Life Sciences, Chongqing Medical University, Chongqing, China; 5 Department of Molecular Biophysics & Biochemistry, Yale University, New Haven, USA; 6 Department of Chemistry, Yale University, New Haven, USA; 7 State Key Laboratory of Medicinal Chemical Biology, College of Pharmacy, Nankai University, Tianjin, China; 8 Tianjin International Joint Academy of Biotechnology and Medicine, Tianjin, China; 9 State Key Laboratory of Oncogenes and Related Genes, Ren Ji Hospital, School of Medicine, Shanghai Jiao Tong University, Shanghai, China; 10 State Key Lab of Biotherapy and Cancer Center, West China Hospital, Sichuan University and Collaborative Innovation Center for Biotherapy, Chengdu, China; 11 State Key Laboratory of Pharmaceutical Biotechnology, Department of Biotechnology and Pharmaceutical Sciences, School of Life Sciences, Nanjing University, Nanjing, China

**Keywords:** Microbiology, Structural biology

## Abstract

Cytoplasmic incompatibility (CI) results when *Wolbachia* bacteria-infected male insects mate with uninfected females, leading to embryonic lethality. “Rescue” of viability occurs if the female harbors the same *Wolbachia* strain. CI is caused by linked pairs of *Wolbachia* genes called CI factors (CifA and CifB). The co-evolution of CifA-CifB pairs may account in part for the incompatibility patterns documented in insects infected with different *Wolbachia* strains, but the molecular mechanisms remain elusive. Here, we use X-ray crystallography and AlphaFold to analyze the CI factors from *Wolbachia* strain *w*Mel called CidA^*w*Mel^ and CidB^*w*Mel^. Substituting CidA^*w*Mel^ interface residues with those from CidA^*w*Pip^ (from strain *w*Pip) enables the mutant protein to bind CidB^*w*Pip^ and rescue CidB^*w*Pip^-induced yeast growth defects, supporting the importance of CifA-CifB interaction in CI rescue. Sequence divergence in CidA^*w*Pip^ and CidB^*w*Pip^ proteins affects their pairwise interactions, which may help explain the complex incompatibility patterns of mosquitoes infected with different *w*Pip strains.

## Abstract (web summary)

*Wolbachia* induced cytoplasmic incompatibility (CI) is caused by linked pairs of genes named *cifA* and *cifB*. Here, authors show that the residues at interfaces of the CidA-CidB complex is crucial for their binding and contribute to the diversity of CI.

## Introduction

*Wolbachia pipientis* is an intracellular bacterium infecting ~40% of all terrestrial arthropod species and certain filarial nematodes^1–3^. They are primarily inherited maternally^4^. Their remarkable success in spreading in arthropod populations is in part driven by the ability of *Wolbachia* to increase the number of infected females^5^, often by acting as reproductive parasites that distort host sex ratios or reproductive outcomes^6^. The most common type of reproductive parasitism is cytoplasmic incompatibility (CI)^6^, which is a type of post-zygotic male sterility characterized by improper paternal chromatin condensation and separation in the first mitosis cycle^7–9^. CI causes *Wolbachia-*infected male insects to be sterile in matings with uninfected females but not similarly infected females (called ‘rescue’), providing a selective advantage for infected females and thus driving *Wolbachia* into the host population^6,10^. Insect species infected by two or more different *Wolbachia* strains can be bidirectional incompatible^11–13^, which causes reproductive isolation of populations infected with different strains^11,14^.

Based on the outcomes of crosses between *Wolbachia-*infected insects, it has been proposed that CI is governed by a modification-rescue system^15,16^. Specifically, a modification activity from *Wolbachia* is postulated to modify sperm during spermatogenesis, and a rescue activity in the infected egg reverses or neutralizes the original sperm modification following fertilization. The existence of both unidirectional and bidirectional incompatibilities in infected insect populations has led to agreement that *Wolbachia* may carry multiple modification and rescue factors^12,13^, but the molecular identity of these factors remained unknown for nearly half a century^17–20^.

Recently, the key factors involved in CI induction and rescue were identified^21–24^. They are known as CI factors or Cifs, which are encoded by pairs of linked genes, *cifA* and *cifB*. Transgenic expression of both *cifA* and *cifB* genes, or *cifB* alone, in the germline of male *Drosophila melanogaster* or *Anopheles gambiae*, induces sterility that is highly similar to CI induced by *Wolbachia*^21,22,25–27^. This embryonic lethality can be rescued by crossing transgenic males with *Wolbachia-*infected females or those with a transgenic *cifA* gene expressed in the germline^22–24^. The *cif* genes have diverged into at least five distinct phylogenetic groups, which are named types I-V^22,28–30^. The degree of similarity and the presence/absence of *cif* gene homologs between *Wolbachia* strains correlates with known patterns of bidirectional incompatibility. For instance, *Wolbachia* strain *w*Ri is able to rescue *w*Mel-induced CI in same-species crosses, probably because these two strains share highly related type-I homologs of the *cifA* gene (99% amino acid identity); however, the reverse is not true: *w*Mel cannot rescue *w*Ri-induced CI, likely due to *w*Ri also encoding a type II gene pair that is much more divergent^12,13,24^. In general, the *cif* genes from bidirectionally incompatible *Wolbachia* pairs are highly divergent, with only 29–68% amino-acid identity^22^. Recent analysis of transgenic *cif* gene expression in *D. melanogaster* is largely consistent with this view^31^.

The *cifA* and *cifB* genes are coevolving^22,30,32^. Protein-binding experiments and expression of *cif* genes in yeast have shown that CifA binds its cognate CifB specifically and rescues CifB-induced yeast growth defects^21,23^. Although the specific roles of *cifA* and *cifB* in CI induction and rescue are still not fully understood, the co-divergence of the *cifA* and *cifB* genes^22,32,33^ has been proposed to be partially responsible for bidirectional incompatibility, possibly by modulating CifA-CifB binding^34,35^.

**Fig. 1:**
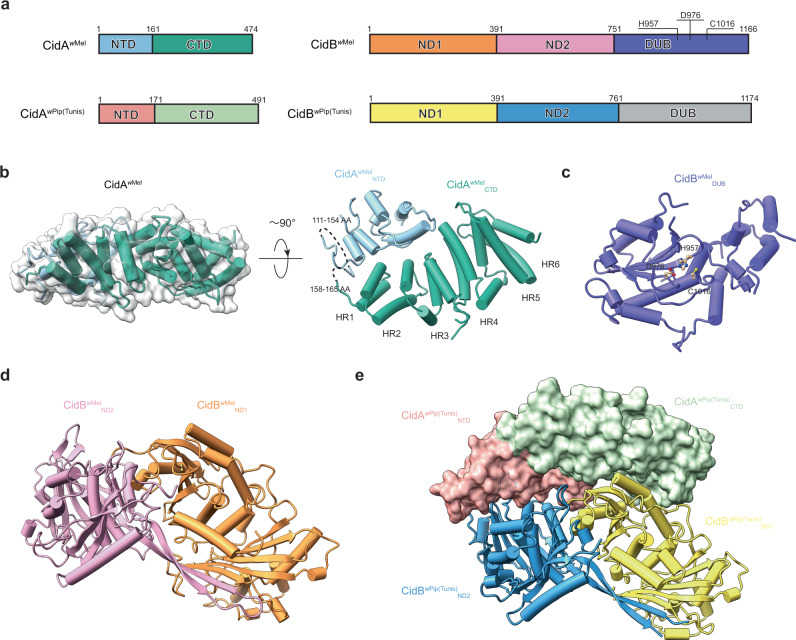
Crystal structures of CidA^*w*Mel^ and CidB^*w*Mel^_DUB_, and a model of CidB^*w*Mel^_ND1-ND2_ reveal the molecular basis for CI. **a** CidA^*w*Mel^ and CidB^*w*Mel^, and CidA^*w*Pip^ and CidB^*w*Pip^ form alternative two-gene CI systems. Each domain is assigned a unique color. **b** CidA^*w*Mel^ contains mostly α-helices. The C-terminal domain contains six HEAT repeats (HR1-HR6). Residues 111-154 and 158-165 are disordered. **c** CidB^*w*Mel^_DUB_ consists of a five-stranded β sheet flanked by α helices on both sides. The active center residues are labeled and shown as balls and sticks. **d** A model for CidB^*w*Mel^_ND1-ND2_ was built with AlphaFold. **e** The crystal structure of the CidA^*w*Pip(Tunis)^-CidB^*w*Pip(Tunis)^_ND1-ND2_ complex. NTD: N-terminal domain; CTD C-terminal domain; ND nuclease domain; DUB deubiquitylase domain; AA amino acids; HR HEAT repeats.

Among the five types of *cif* genes, only type I and certain type V *cifB* genes encode a deubiquitylase (DUB) domain^21^. (Hereafter, we will use “Cif” for the entire set of CI factors and “Cid” for the deubiquitylase family and primarily focus on the type I Cid protein family.) The *cidA and cidB* genes from *Wolbachia* strains *w*Mel (hereafter *cidA*^*w*Mel^ and *cidB*^*w*Mel^) and *w*Pip (hereafter *cidA*^*w*Pip^ and *cidB*^*w*Pip^) are among the most well studied *cif* genes (Fig. 1a). These *Wolbachia* strains are expected to be incompatible, as shown by crosses between trans-infected *Aedes albopictus* mosquitoes^36^. Unlike the *w*Mel strain, *Wolbachia w*Pip strains found in different populations of *Culex pipiens* are highly diversified. Many of them carry several polymorphic copies of the *cidA*^*w*Pip^ and *cidB*^*w*Pip^ genes, which may function as independent modification and rescue factors and associate with the diverse CI phenotypes in *w*Pip-infected *C. pipiens*^32,37^. Thus, the Cid proteins can be used as a model to study how the diversification of *cidA*^*w*Pip^ and *cidB*^*w*Pip^ affects protein-protein interactions and how it is related to CI induction and rescue.

Here, we report crystal structures for CidA^*w*Mel^ and the DUB domain of CidB^*w*Mel^ and build a model of the CidA^*w*Mel^-CidB^*w*Mel^ complex using AlphaFold-Multimer^38^, which is validated and confirmed by comparison to a crystal structure of a homologous CidA^*w*Pip^-CidB^*w*Pip^ complex. Substituting the CidA^*w*Mel^ residues at the CidB-binding interfaces with those of CidA^*w*Pip^ enables it to bind CidB^*w*Pip^ and rescues CidB^*w*Pip^-induced yeast growth defects, which suggests a role of *cid* gene co-evolution in modulating CidA-CidB binding specificity. Analysis using the crystal structures of the CidA^*w*Pip^-CidB^*w*Pip^ complex further shows that sequence divergence in *Wolbachia w*Pip may result in complex binding patterns between Cid^*w*Pip^ protein variants. These results provide a solid base for future investigations of CI mechanism and shed light on how co-evolution of CidA-CidB pairs modulates their interactions and bidirectional CI.

## Results

### Crystal structures of CidA^*w*Mel^ and CidB^*w*Mel^_DUB_, and a model of CidB^*w*Mel^_ND1-ND2_

We determined the crystal structure of CidA^*w*Mel^ to 2.75 Å resolution (Supplementary Table 1). CidA^*w*Mel^ is made up largely of α-helices (Fig. 1b). Residues 111-154 and 158-165 are disordered and cannot be modeled, which divide CidA^*w*Mel^ into an N-terminal domain (NTD, residues 1 to 161) (see below for why we include these disordered residues as part of the N-terminal domain) and a C-terminal domain (CTD, residues 162 to 422) (Fig. 1b). The C-terminal domain folds into a twisted set of six HEAT repeats, a structural motif that primarily mediates protein-protein interactions^39^ (Fig. 1b).

CidB^*w*Mel^ was expressed poorly in *Escherichia coli*, making direct structure determination difficult. We used a “divide-and-conquer” strategy to obtain structural information for each of its domains separately. CidB^*w*Mel^ consists of a region, which is predicted to contain two PD-(D/E)XK (pseudo) nuclease domains (residue 1-751; hereafter CidB^*w*Mel^_ND1-ND2_, ND: nuclease domain) and a C-terminal deubiquitylase (DUB) domain. An active DUB domain is required for inducing a CI-like phenotype in transgenic *D. melanogaster* for *cid* operons^21^. We determined the crystal structure of the CidB^wMel^ DUB domain to 1.85 Å resolution (Supplementary Table 1 and Fig. 1c). Similar to the structures of other proteases in the CE clan/Ulp1-like protease family^40,41^, the DUB core consists of a five-stranded β sheet flanked by α helices on both sides. His957, Asp976, and Cys1016 form the catalytic triad (Fig. 1c). Three variable regions (VRs), a constant region (CR) and a C-terminal accessory domain may account for the S1 ubiquitin-binding interface^41^ (Supplementary Fig. 1a-d).

A model for CidB^*w*Mel^_ND1-ND2_ (Fig. 1d) was built with the AlphaFold^42^ program. Although the predicted structural model has an average pLDDT score as high as 83.190, which confirms its quality (pLDDT > 70 indicates the backbone prediction is correct^43^), we wanted to validate this model further.

**Fig. 2:**
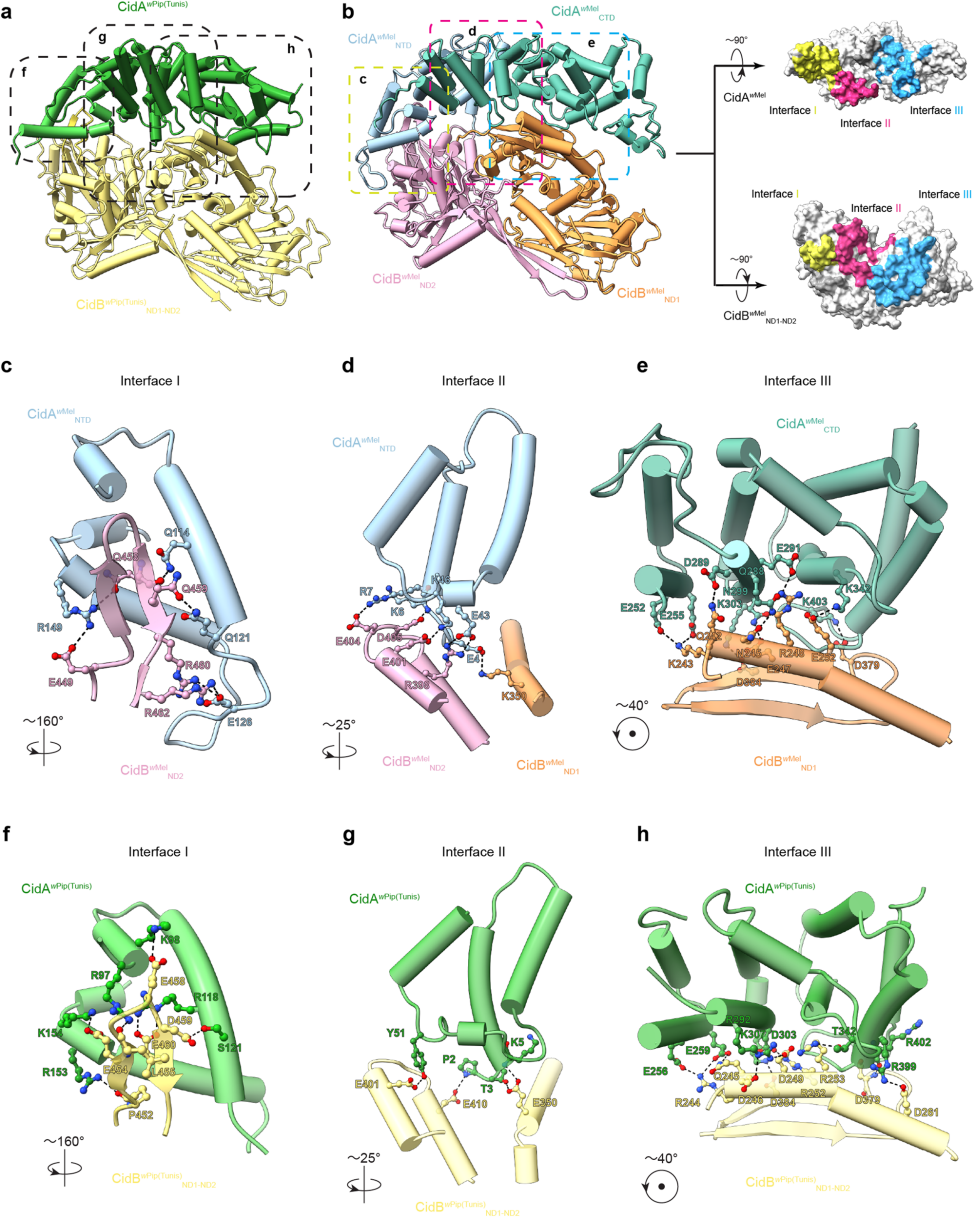
CidA and CidB interact through a large conserved tripartite interface. **a** The structure of the CidA^*w*Pip(Tunis)^-CidB^*w*Pip(Tunis)^_ND1-ND2_ complex. CidA^*w*Pip(Tunis)^ binds CidB^*w*Pip(Tunis)^_ND1-ND2_ through three regions (Interface I, II, and III). **b** A structural model of CidA^*w*Mel^ in complex with CidB^*w*Mel^_ND1-ND2_ was generated by AlphaFold-Multimer. The tripartite interface between CidA^*w*Mel^ and CidB^*w*Mel^_ND1-ND2_ is shown in yellow, magenta and cyan for Interface I, II, and III, respectively. **c**–**e** Each interface of the CidA^*w*Mel^-CidB^*w*Mel^_ND1-ND2_ complex involves a pair of structural motifs. Representative residues directly involving in the interaction are labeled and shown as balls and sticks. **f**–**h** The structural motifs at Interface I, II and III of the CidA^*w*Pip(Tunis)^-CidB^*w*Pip(Tunis)^_ND1-ND2_ complex are shown, with residues directly involving in the interaction labeled. NTD N-terminal domain; CTD C-terminal domain; ND nuclease domain.

CidB^*w*Mel^_ND1-ND2_ shares sequence homology with other CifB molecules^34^. Of note, the ND1-ND2 region of CidB^*w*Pip^_I(b/2) (a CidB^*w*Mel^ ortholog from the *Wolbachia* strain *w*Pip Tunis line^30^, hereafter CidB^*w*Pip(Tunis)^_ND1-ND2_) expressed well in *E. coli* and shares a sequence identity of 71% with CidB^*w*Mel^_ND1-ND2_ (Supplementary Fig. 2). We successfully determined a crystal structure of CidB^*w*Pip(Tunis)^_ND1-ND2_ in complex with CidA^*w*Pip(Tunis)^ (specifically CidA^*w*Pip^_I(γ/2)^32^) (Supplementary Table 1, Figs. 1e and 2a). The structure of CidB^*w*Pip(Tunis)^_ND1-ND2_ could be used for comparison with CidB^*w*Mel^_ND1-ND2_ modeled by AlphaFold^42^.

The structure of CidB^*w*Pip(Tunis)^_ND1-ND2_ is very similar to the model for CidB^*w*Mel^_ND1-ND2_ (Supplementary Fig. 3a). Moreover, the residues that are varied among CidB homologs are mainly located at the surfaces of the protein (Supplementary Fig. 4a), which should not affect protein folding. Thus, the model for CidB^*w*Mel^_ND1-ND2_ is likely to be accurate. Since the *cif* genes from *w*Mel have been well characterized, we will mainly focus on the structures and functional analysis of the CidA^*w*Mel^-CidB^*w*Mel^_ND1-ND2_ pair in the following sections. The results from the CidA^*w*Pip(Tunis)^-CidB^*w*Pip(Tunis)^ system will be mentioned when necessary.

CidB^*w*Mel^_ND1-ND2_ consists of two NDs; ND1 comprises residues 1-391 and ND2 comprises residues 392-751 (Fig. 1d). The ND1 and ND2 domains of CidB^*w*Mel^ interact through an extensive interface burying a surface area of ~2200 Å^2^. This interface is highly similar to that of CidB^*w*Pip(Tunis)^ (Supplementary Fig. 3b-c) and the residues involving in ND1-ND2 interaction are highly conserved among CidB homologs (Supplementary Fig. 3d-f). Interestingly, in addition to the (inactive) PD-(D/E)XK modules predicted by sequence homology analysis (residues 277-375 and 607-730)^21,28–30^, the structural model of CidB^*w*Mel^ shows that each ND has an additional more divergent PD-(D/E)XK module (residues 30-137 and 431-523) (Supplementary Fig. 5). None of these modules present the key canonical catalytic residues E-D-E-K^23^ (the putative corresponding residues are P33, K77, S92, S94 in module I, K279, Y311, N330, T332 in module II, F435, K475, I491, D493 in module III and K609, G663, V680, G682 in module IV based on structural analysis (Supplementary Fig. 5)). These modules may play structural roles or support an alternative catalytic activity.

### CidA^*w*Mel^ binds CidB^*w*Mel^ through a large distinct interface

We built a model for the CidA^*w*Mel^-CidB^*w*Mel^_ND1-ND2_ complex using AlphaFold-Multimer^38^ (Fig. 2b). The predicted end-to-end structure has a ranking score (TM-Score) as high as 0.8568 with a ptm score (intra-chain quality) of 0.86436 and an iptm score (interface score) of 0.85498. This indicates our prediction is highly reliable both for the protein themselves and the interface between them. The obtained end-to-end model was further optimized by Molecular Dynamics (MD) simulations. Two independent trajectories were performed and both achieved equilibrium within the first 100 ns and were then trapped in optima in the next 100 ns. The Root-Mean-Square Deviation (RMSD) for one of the simulation trajectories was recorded (Supplementary Fig. 6a). Trajectory cluster analysis was performed and the most stable binding conformation was extracted from the largest cluster of the trajectory as the final binding complex of CidA^*w*Mel^-CidB^*w*Mel^_ND1-ND2_. The complex also predicts many hydrogen bonds and salt bridges between CidA^*w*Mel^ and CidB^*w*Mel^_ND1-ND2_ at the three interfaces (Fig. 2c–e) and only 0.57% of the residues are Ramachandran outliers (Supplementary Fig. 6b), which demonstrated the side chains of the predicted model have been reliably predicted. Therefore, we believe that the model of the CidB^*w*Mel^_ND1-ND2_ and its complex with CidA^*w*Mel^ is reasonable.

The interface between CidA^*w*Mel^ and CidB^*w*Mel^_ND1-ND2_ can be divided into three regions (Fig. 2b); each region mainly involves a pair of structural motifs. At the first region (Interface I), the helices consisting of the residues 93-158 of CidA^*w*Mel^ interact with a loop in CidB^*w*Mel^_ND1-ND2_ (residues 448-462) through a network of hydrogen bonds and salt bridges (Fig. 2c). Interestingly, the region of CidA^*w*Mel^ which is disordered in the absence of CidB^*w*Mel^ (residue 111-154 and 158-165) is modeled to form a helix bundle upon complex formation (Figs. 1b and 2c), consistent with its role in mediating CidA^*w*Mel^-CidB^*w*Mel^_ND1-ND2_ interaction. These residues are part of the N-terminal domain. Interface II is formed by the helices at the N-terminus of CidA^*w*Mel^ (residues 2-60) and helices from both NDs of CidB^*w*Mel^_ND1-ND2_ (residues 337-353 and 395-418) (Fig. 2d). Interface III involves multiple HEAT repeats in CidA^*w*Mel^ and a cross-cutting helix (residues 241-264) in CidB^*w*Mel^_ND1-ND2_ that is bolstered by two β strands and their connecting loop (residues 365-387) to stabilize the interaction (Fig. 2e). Interestingly, although the model of CidA^*w*Mel^-CidB^*w*Mel^_ND1-ND2_ complex is very similar to the structure of CidA^*w*Pip(Tunis)^-CidB^*w*Pip(Tunis)^_ND1-ND2_ (Fig. 2a, b), the residues involving in interaction at the three interfaces are very different between the two complexes (Fig. 2c–h), which could help explain their cognate-specific binding. (The residues at the interface are well resolved in CidA^*w*Pip(Tunis)^-CidB^*w*Pip(Tunis)^_ND1-ND2_ with good electron density (Supplementary Fig. 7)).

Pulldown experiments and yeast growth assays were used to validate these structural models. As a model system, it has been demonstrated that expression of *cidB* genes in the yeast S*accharomyces cerevisiae* inhibits cell growth, but growth is at least partially restored if the cognate *cidA* gene is coexpressed, resembling CI induction and rescue in insects^21,23^. Substituting the CidA^*w*Pip(Tunis)^ residues at its CidB-binding interface with the corresponding ones from CidA^*w*Mel^ abolished its ability to bind CidB^*w*Pip(Tunis)^_ND1-ND2_ and neutralize the CidB^*w*Pip(Tunis)^-induced growth defect in yeast (Supplementary Fig. 8). Thus, the structure of the CidA^*w*Pip(Tunis)^-CidB^*w*Pip(Tunis)^_ND1-ND2_ complex accurately captures the CidA-CidB interaction mode and is biologically relevant. However, due to the poor expression of CidB^*w*Mel^_ND1-ND2_ in *E. coli* and the low toxicity of CidB^*w*Mel^ in yeast, we could not directly validate the model of the complex by pulldown or yeast growth analysis. To test the reliability of this model, we rationally designed a set of mutations based on the model to demonstrate that the interaction mode is conserved between the *w*Pip and *w*Mel CidA-CidB pairs (see below).

### CidA-CidB binding specificity is determined by residues at their interfaces

**Fig. 3:**
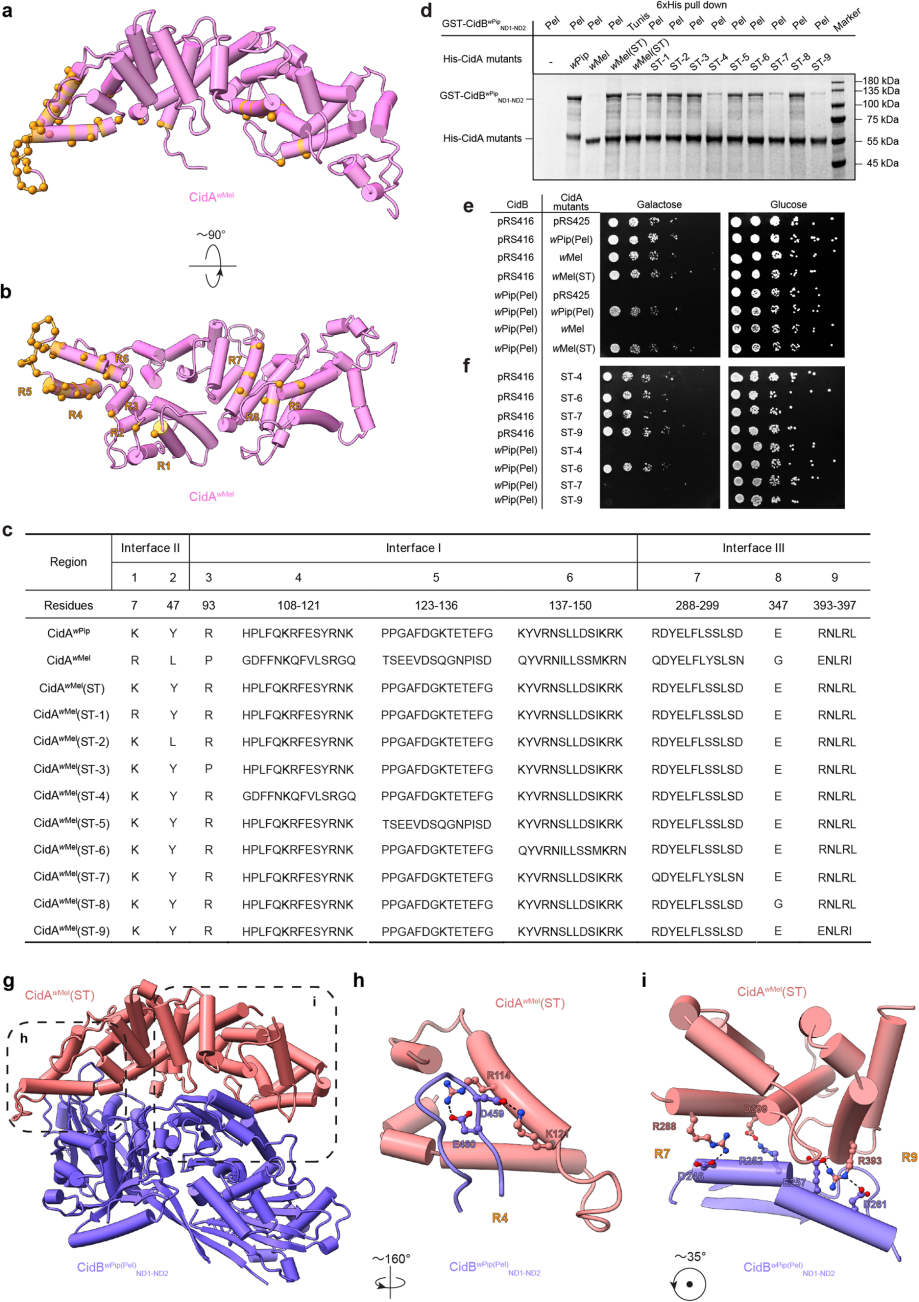
Mutagenesis with binding and yeast growth assays reveal how residues at the three interfaces determine CidA binding specificity. **a**, **b** CidA^*w*Mel^(ST) is a chimera with the body of CidA^*w*Mel^ (pink) and interfacial residues from CidA^*w*Pip(Tunis)^. The locations of the mutated residues are shown in orange on the CidA^*w*Mel^ structure. **c** The substituted residues in CidA^*w*Mel^(ST) are divided into nine regions (R) and reversed back to those of CidA^*w*Mel^, individually, to create CidA^*w*Mel^(ST-1) through CidA^*w*Mel^(ST-9). **d** CidA^*w*Mel^(ST) does not bind wild-type CidB^*w*Mel^ but binds CidB^*w*Pip(Pel)^ to a similar extent as CidA^*w*Pip(Pel)^. Regions 4, 7, and 9 play important roles in binding. The experiment was repeated three times independently with similar results obtained. One representative is shown. **e** CidA^*w*Mel^(ST) is able to rescue yeast from CidB^*w*Pip(Pel)^-induced lethality. **f** CidA^*w*Mel^(ST-4), CidA^*w*Mel^(ST-7) and CidA^*w*Mel^(ST-9), which do not bind CidB^*w*Pip(Pel)^, also fail to suppress CidB^*w*Pip(Pel)^-induced yeast growth defects. **g** The crystal structure of the CidA^*w*Mel^(ST)-CidB^*w*Pip(Pel)^_ND1-ND2_ complex is similar to the model of the CidA^*w*Mel^-CidB^*w*Mel^_ND1-ND2_ complex. Representative residues directly involved in the CidA^*w*Mel^(ST) and CidB^*w*Pip(Pel)^_ND1-ND2_ interactions at Interface (**h**) I and (**i**) III are labeled. ST substituted; ND nuclease domain. Source data for panels (**d**) and (**e**) are provided as a Source Data file.

Until now, only CifA and CifB proteins expressed from the same operon have been shown to interact^21,23,35^. The specific interactions between CifA and CifB have been proposed to play a role in CI induction and/or rescue, depending on different CI models. We substituted multiple residues at the binding interface of CidA^*w*Mel^ with the corresponding region of CidA^*w*Pip(Tunis)^ (Fig. 3a–c) and investigated if the chimeric construct (named CidA^*w*Mel^(ST)) could now bind CidB^*w*Pip(Tunis)^_ND1-ND2_. Their interaction, while detectable, was weak, probably because the residues substituted are not the major binding determinants for interaction between CidA^*w*Pip(Tunis)^ and CidB^*w*Pip(Tunis)^_ND1-ND2_ (Fig. 3d). Interestingly, CidA^*w*Mel^(ST) could bind a closely related CidB^*w*Pip^_ND1-ND2_ from *Wolbachia* strain *w*Pip(Pel) (hereafter CidB^*w*Pip(Pel)^_ND1-ND2_) to an extent similar to the binding of the cognate CidA^*w*Pip(Pel)^ (Fig. 3d, lane 2 vs. lane 4). By contrast, CidA^*w*Mel^ had been shown previously to be unable to bind CidB^*w*Pip(Pel)^ (Ref. 21). CidB^*w*Pip(Pel)^_ND1-ND2_ only differs from CidB^*w*Pip(Tunis)^_ND1-ND2_ by 30 residues (Supplementary Fig. 9). We used CidB^*w*Pip(Pel)^ for further biochemical investigation.

Agreeing with the pulldown experiments, yeast growth defects induced by CidB^*w*Pip(Pel)^ could be rescued by both CidA^*w*Mel^(ST) and CidA^*w*Pip(Pel)^, but not CidA^*w*Mel^ (Fig. 3e) even though the expression levels for CidA^*w*Mel^ and CidA^*w*Mel^(ST) were similar (Supplementary Fig. 10). These data clearly show the importance of the interfacial residues (Fig. 3e) in determining binding specificity. They also support the accuracy of our CidA^*w*Mel^-CidB^*w*Mel^_ND1-ND2_ model.

To locate the critical regions that determine binding specificity, the substituted interfacial residues of CidA^*w*Mel^(ST) were divided into nine groups/regions based on their location in the primary sequence and three-dimensional structure. Each region was individually reverted back to the original sequence in CidA^*w*Mel^ (Fig. 3b, c), resulting in CidA^*w*Mel^(ST-1) to CidA^*w*Mel^(ST-9). (Regions 1, 2 are at Interface II. Regions 3, 4, 5, 6 are at Interface I. Regions 7, 8, 9 are at Interface III.) CidA^*w*Mel^(ST-4), CidA^*w*Mel^(ST-7) and CidA^*w*Mel^(ST-9) showed reduced binding affinity to CidB^*w*Pip(Pel)^_ND1-ND2_ (Fig. 3d). They also failed to rescue CidB^*w*Pip(Pel)^-induced yeast growth defects (Fig. 3f). (These CidA variants were expressed at similar levels. Interestingly, however, co-expression of CidA^*w*Mel^ variants with CidB^*w*Pip(Pel)^ in yeast did strongly enhance levels of the latter protein but only if they were able to bind to it. (Supplementary Fig. 10)) Thus, these specific residues at the binding interface are particularly important in determining the binding specificity of CidA^*w*Mel^.

### Crystal structure of CidA^*w*Mel^(ST)-CidB^*w*Pip(Pel)^_ND1-ND2_ explains interaction specificity

We next determined the crystal structure of the CidA^*w*Mel^(ST)-CidB^*w*Pip(Pel)^_ND1-ND2_ complex (Supplementary Table 1 and Fig. 3g). The structure of the complex was very similar to that of CidA^*w*Pip(Tunis)^-CidB^*w*Pip(Tunis)^_ND1-ND2_ (RMSD: 0.8 Å over 691 Cα atoms). This structure revealed the interacting residues at the CidA^*w*Mel^(ST)-CidB^*w*Pip(Pel)^_ND1-ND2_ interface (Supplementary Fig. 11; electron density for representative residues shown in Supplementary Fig. 7) and explained why certain CidA^*w*Mel^(ST) variants show reduced binding to CidB^*w*Pip(Pel)^_ND1-ND2_. For example, at region 4, residues R114 and K121 of CidA^*w*Mel^(ST) interact electrostatically with residues E460 and D459 of CidB^*w*Pip(Pel)^_ND1-ND2_, respectively (Fig. 3h). In CidA^*w*Mel^(ST-4), residues 114 and 121 were reverted back to the corresponding residues of CidA^*w*Mel^, which are both glutamines (Fig. 3c). These residues are not charged and may lead to the reduced binding of CidA^*w*Mel^(ST-4) to CidB^*w*Pip(Pel)^_ND1-ND2_ (Fig. 3d). Similarly, at region 7, residues R288 and D299 of CidA^*w*Mel^(ST) have charge complementarity with D246 and R252 of CidB^*w*Pip(Pel)^_ND1-ND2_, respectively (Fig. 3i). In the CidA^*w*Mel^(ST-7) revertant, residues 288 and 299 are glutamine and asparagine, respectively (Fig. 3c), which can no longer form salt bridges with D246 and R252 of CidB^*w*Pip(Pel)^_ND1-ND2_. Finally, at region 9, residue R393 of CidA^*w*Mel^(ST) interacts with residues E257 and D261 of CidB^*w*Pip(Pel)^_ND1-ND2_ through salt bridges (Fig. 3i). In CidA^*w*Mel^(ST-9), residue 393 is glutamic acid (Fig. 3c), which cannot bind to E257 and D261 of CidB^*w*Pip(Pel)^_ND1-ND2_ due to electrostatic repulsion. The crystal structure clearly explains the reduced binding of CidA^*w*Mel^(ST-4), CidA^*w*Mel^(ST-7) and CidA^*w*Mel^(ST-9) to CidB^*w*Pip(Pel)^_ND1-ND2_ (Fig. 3d), which closely parallels their loss of function in yeast growth rescue assays (Fig. 3e, f).

### Natural sequence variations affect CidA^*w*Pip(Tunis)^-CidB^*w*Pip(Tunis)^ binding

**Fig. 4:**
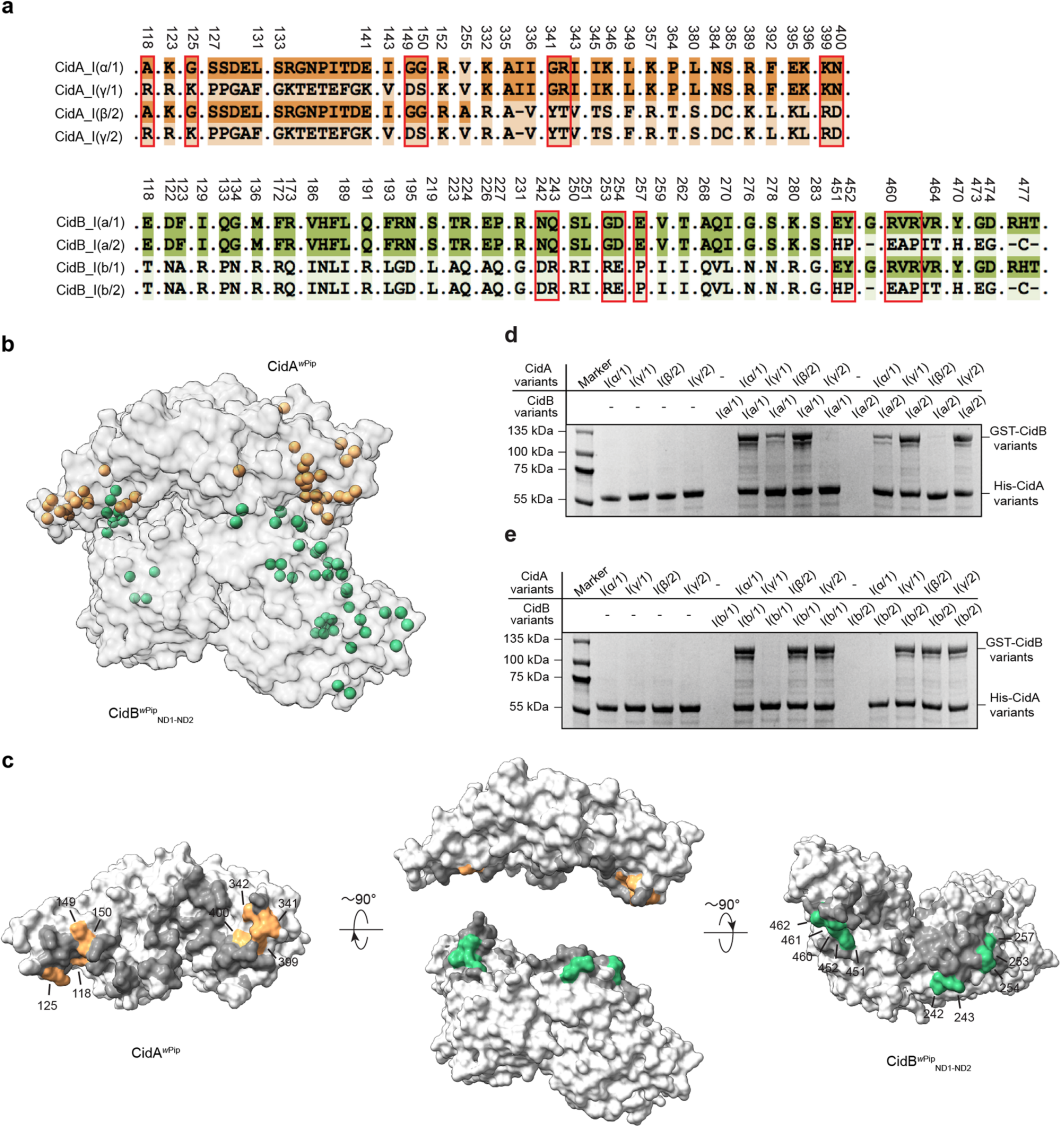
Sequence variations modulate interactions between natural CidA^*w*Pip^ and CidB^*w*Pip^ alleles. **a** Sequence alignment shows that residues at certain positions are different among the CidA^*w*Pip^ and CidB^*w*Pip^ variants. The residues that are the same among the variants are not shown. The varied residues which are located at the binding interfaces are boxed. The numbers above sequence alignment were based on the sequences of CidA and CidB from *w*Pip Tunis. **b** The locations of the varied residues are shown as spheres on the structure of the CidA^*w*Pip(Tunis)^-CidB^*w*Pip(Tunis)^_ND1-ND2_ complex. **c** The varied residues which are located at the binding interfaces are colored and labeled. Other residues at the binding interfaces are shown in gray. **d**, **e** His-tagged CidA variants were used to pull down GST-tagged CidB variants. The proteins were detected by Coomassie Blue staining. These experiments were repeated three times independently with similar results obtained. One representative is shown. ND nuclease domain. Source data for panels (**d**) and (**e**) are provided as a Source Data file.

The *w*Pip *cidA-cidB* operon is duplicated and diversified extensively among CI-inducing *w*Pip strains. Interestingly, however, only residues at certain positions are different among the CidA^*w*Pip^ and CidB^*w*Pip^ variants^32^ (Fig. 4a, b). If the varied residues are at the CidA^*w*Pip^-CidB^*w*Pip^ binding interface, they may affect the interaction of these proteins and play a role in CI induction and/or rescue specificity. The *w*Pip Tunis line belongs to group I of *w*Pip (*w*PipI, grouping of *w*Pip strains is based on phylogenetic analysis of seven *Wolbachia* genes)^32,44^, so we focused on the CidA^*w*Pip^ and CidB^*w*Pip^ variants from this group (hereafter, CidA^*w*Pip^_I and CidB^*w*Pip^_I). (As mentioned before, CidA^*w*Pip(Tunis)^ and CidB^*w*Pip(Tunis)^ are CidA^*w*Pip^_I(γ/2) and CidB^*w*Pip^_I(b/2), respectively.)

Among the positions that show divergence (Fig. 4b), residues 118, 125, 149, 150, 341, 342, 399, and 400 of CidA^*w*Pip^ and residues 242, 243, 253, 254, 257, 451, 452, 460, 461, and 462 of CidB^*w*Pip^ map to the binding interface of the two proteins (Fig. 4c). These variations create distinct interaction surfaces for each of the four known CidA^*w*Pip^_I variants (Fig. 4a). Specifically, CidA^*w*Pip^ variants I(α/1) and I(β/2) have the same residues A118, G125, G149 and G150; the corresponding residues in variants I(γ/1) and I(γ/2) are R118, K125, D149, and S150 (Fig. 4a). CidA^*w*Pip^ variants I(α/1) and I(γ/1) both share residues G342, R343, K400, and N401, which correspond to residues Y341, T342, R399, and D400 in variants I(β/2) and I(γ/2). Similarly, each of the four CidB^*w*Pip^_I variants has a unique combination of residues at the CidA-binding interface (Fig. 4a). Pulldown experiments were used to investigate the interactions between the CidA^*w*Pip^ variants and the CidB^*w*Pip^ variants. His-tagged CidA variants I(α/1), I(γ/1), I(β/2), and I(γ/2) cannot pull down GST-tagged CidB variants I(b/2), I(b/1), I(a/2), and I(a/1), respectively (Figs. 4d, e), which supports the hypothesis that natural sequence variations affect binding interactions between CidA^*w*Pip^ and CidB^*w*Pip^ from different strains. If binding is required for CidA to rescue CidB-induced defects, this sequence diversity may at least partially explain the complex bidirectional incompatibility patterns in crosses between different *C. pipiens* lines (discussed below).

## Discussion

Here, we have presented structural, biochemical and functional studies on the *Wolbachia* CI factors CidA^*w*Mel^ and CidB^*w*Mel^, providing a solid foundation for investigation of the molecular functions of the Cid proteins in CI. These proteins are used here as a model to investigate the evolution of CidA-CidB interactions and their biological impact. Our structure-based mutagenesis has led to a clear demonstration of noncognate CifA-CifB binding, which suggests how sequence changes specifically at the interfaces between these proteins could lead to the complex incompatibility relationships that can be observed in the wild^32^.

The Cid systems from *Wolbachia* strains *w*Mel and *w*Pip are evolutionarily related, but these strains reside in different clades or supergroups (supergroups A and B, respectively). The two strains are expected to be incompatible based on crosses with trans-infected *Ae. albopictus* mosquitoes^36^. Here, we show that the CidA-CidB pairs from these *Wolbachia* strains share the same interaction mode but have different residues at their binding interfaces. These residues are critical for determining their binding specificity. CidA^*w*Mel^(ST), a rationally designed chimeric construct containing the scaffold of CidA^*w*Mel^ and the interfacial residues of CidA^*w*Pip^, binds CidB^*w*Pip(Pel)^ and rescues CidB^*w*Pip(Pel)^-induced growth defects in yeast. It proves that CidA variants from different *Wolbachia* strains use the same pathway to carry out their function.

To further evaluate the conservation and evolutionary relationship of CidA and CidB found in different *Wolbachia* strains, the conservation scores of CidA and CidB residues were calculated by the ConSurf server and mapped onto their structures^45–47^. The residues in the core of the proteins tend to be highly conserved. In contrast, the residues located at the surface are more variable (Supplementary Fig. 4a-b). Many of the varied residues are located at the CidA-CidB binding interfaces (Supplementary Fig. 4c). (Although several key residues of CidA at interface III are identical among its homologs, their interaction with CidB cannot be maintained due to residue variations at interface III of CidB.) This agrees with previous hypotheses that the CidA and CidB interfaces are co-evolving^32,35^, which could modulate binding between CidA and CidB proteins and lead to incompatibility.

*Wolbachia* strains infecting *C. pipiens* usually contain more than one copy of the *cidA* and *cidB* genes^32^, which has been proposed to be responsible for the very complex incompatible patterns documented in *C. pipiens* infected with these strains. We showed here that the natural sequence variations in CidA^*w*Pip^ and CidB^*w*Pip^ proteins affects their binding specificity. The expression of multiple CidA^*w*Pip^ and CidB^*w*Pip^ variants in one *Wolbachia* strain could lead to a mix-and-match type of binding and may be one reason for the very complex crossing results. The presence of *cidB_*IV(2) variants was associated with the incompatible phenotype of mosquitoes infected with group IV *w*Pip when crossing with those infected with *w*Pip from other groups^32,37^. Indeed, the CidB*_*IV(2) variants have unique residues at interface I (including residues 451, 452, 460, 461 and 462) compared to the CidB of the other groups. These differences may impair the binding of the CidB*_*IV(2) variants to the CidA variants of the other groups and lead to incompatibility.

The crystal structure of CidB^*w*Mel^_DUB_ provides a solid structural basis for further investigation of the molecular targets and function of CidB deubiquitylase family proteins. Given that bacteria do not possess a ubiquitin-conjugation system, the presence of a DUB domain in CidB strongly suggests that this *Wolbachia* protein functions within the host^21,40^. Indeed, CidB proteins have been proposed to target nuclear-protein import and protamine-histone exchange factors^48^. CidB^*w*Mel^_DUB_ has three variable regions and a C-terminal region different from other bacterial proteases in the CE-clan/Ulp1-like protease family, which could be responsible for its substrate preference for ubiquitin over other ubiquitin-like molecules and for Lys63-linked ubiquitin chains^21,40,41^.

In different models that try to account for how Cif proteins induce and rescue CI, CifA-CifB interaction is suggested to play different roles. In one, which is named the “2-by-1” model, CifB is suggested to have an ancillary role by modulating CifA stability or activity in the male germline^25^. By contrast, in the “toxin-antidote” model, specific CifA-CifB interaction in the egg has been proposed to be essential for CifA to rescue CifB-induced CI^32,35^. Two major differences between these models are where CifA and CifB interact (in male or female) and whether the interaction is involved in CI induction or rescue. Our structures of CidA-CidB complexes and attempts to modulate their binding specificity provide a way to rationally design CifA and CifB mutants with desired binding attributes. Analysis of these variants will help distinguish between these and other CI models.

## Methods

### DNA manipulation

The genes coding for CidA, the variants of CidA, and CidB of *w*Pip(Tunis), *w*Pip(Pel) and *w*Mel were synthesized at the Beijing Genomics Institute (BGI China). These sequences were codon-optimized for expression in *E. coli* by BGI. For crystallization, CidA^*w*Pip(Tunis)^, CidB^*w*Pip(Tunis)^_ND1-ND2_ (residues 1-761), CidB^*w*Pip(Pel)^_ND1-ND2_ (residues 1-761) and CidA^*w*Mel^ genes were subcloned into the pET-22b(+) vector using restriction sites *Nde*I and *Xho*I, encoding proteins with a C-terminal His-tag. CidB^*w*Mel^_DUB_ (residues 797-1128) gene was subcloned into the pGEX-6p-1 vector possessing an N-terminal glutathione-S-transferase (GST) tag using restriction sites *Bam*HI and *Xho*I.

For in vitro pull-down experiments, CidA variants were the same as those used for crystallization. A pET28a-GST vector was made where the GST coding sequence was inserted between restriction sites *Nco*I and *Bam*HI on pET28a. The CidB^*w*Pip(Tunis)^_ND1-ND2_ and CidB^*w*Pip(Pel)^_ND1-ND2_ genes were cloned into the pET28a-GST expression vector between restriction sites *Eco*RI and *Xho*I so that the proteins expressed had an N-terminal GST-tag.

For yeast growth analysis, DNA fragments were subcloned from *E. coli* vectors by restriction digest or PCR amplification and ligated into yeast expression vectors. The 2-micron plasmid pRS425GAL1 *(LEU2)* utilizing a *GAL1* promoter was used for galactose-induced CifA expression in yeast and the low-copy vector pRS416GAL1 *(URA3)* was used for galactose-induced CifB expression.

Primers used to generate CifA or CifB expression plasmids are summarized in Supplementary Table 2. All plasmids were verified by sequencing (Sangon Biotech, China).

### Protein expression and purification

All proteins were expressed in* E. coli* (BL21 (DE3) strain). Briefly, *E. coli* transformed with an expressing plasmid was cultured in Luria broth (LB) at 37 °C to an optical density (OD_600_) of 0.6. Overexpression of the recombinant proteins was induced by adding isopropyl-β-D-thiogalactopyranoside (IPTG) to a final concentration of 0.5 mM at 16 °C for 16–18 h.

The harvested bacteria overexpressing CidA^*w*Mel^ (or CidA variants) were resuspended in a lysis buffer (20 mM Tris-HCl, pH 8.0, 300 mM NaCl, 10 mM imidazole, 10% glycerol) and lysed via a high-pressure homogenizer at 4 °C. The lysate was centrifugated at 26500 × g for 30 min at 4 °C. After centrifugation, the supernatant was loaded onto a Ni-NTA column (GE Healthcare, USA). The column was washed using a lysis buffer supplemented with 50 mM imidazole and eluted using a lysis buffer supplemented with 500 mM imidazole. The eluted protein was diluted with buffer A (20 mM Tris-HCl, pH 8.0, 5 mM DTT) and further purified by anion-exchange chromatography (Hi Trap Q HP 5 mL, GE Healthcare, USA), using a linear gradient of 0%-40% mixture of buffer A and buffer B (20 mM Tris-HCl, pH 8.0, 1 M NaCl, pH 8.0, 5 mM DTT). Finally, the protein was purified by gel-filtration chromatography (Superdex 200 10/300 GL, GE Healthcare, USA), using a buffer containing 20 mM Tris-HCl, pH 8.0, 150 mM NaCl, 5 mM DTT.

To obtain the CidA^*w*Pip(Tunis)^-CidB^*w*Pip(Tunis)^_ND1-ND2_ and CidA^*w*Mel^(ST)-CidB^*w*Pip(Pel)^_ND1-ND2_ complexes, bacterial cells expressing each component of the specific complex were mixed and co-lysed in the lysis buffer containing 20 mM Tris-HCl, pH 8.0, 0.3 M NaCl, 10 mM imidazole, 10 μg/mL DNase 1, 10 μg/mL RNase A and 10% glycerol. The complex were further purified by running cleared lysate sequentially through Ni-NTA affinity, anion-exchange and size exclusion chromatography using the same columns and buffers as described for CidA^*w*Mel^.

The CidB^*w*Mel^_DUB_ protein was purified following a similar procedure, using affinity, anion-exchange and size exclusion chromatography. CidB^*w*Mel^_DUB_ has a GST-tag. Instead of using Ni-NTA affinity chromatography, GST affinity chromatography was used. The harvested bacteria overexpressing CidB^*w*Mel^_DUB_ were resuspended in a lysis buffer (20 mM Tris-HCl, pH 8.0, 150 mM NaCl, 5% glycerol) and lysed via a high-pressure homogenizer at 4 °C. The lysate was centrifugated at 26500 × g for 30 min at 4 °C. After centrifugation, the supernatant was loaded onto a glutathione-Sepharose column (GE Healthcare, USA). The GST tag was removed by incubating the loaded column with PreScission protease overnight at 4 °C. The CidB^*w*Mel^_DUB_ protein was further purified through anion-exchange and size exclusion chromatography using the same columns and buffers as described above.

Selenomethionine (SeMet)-derivatized proteins were expressed by bacteria growing in M9 SeMet medium supplemented with 100 mg/L L-selenomethionine. The purification procedure of SetMet-derivated proteins was the same as mentioned above for the native.

### Crystallization, data collection, and structure determination

All crystals were grown by the microbatch-under-oil method unless otherwise specified^49^. CidA^*w*Mel^ was crystallized at 16 °C by mixing 1 µL protein (5 mg/mL) with 1 µL crystallization buffer containing 0.2 M Sodium phosphate monobasic monohydrate, 20% w/v Polyethylene glycol 3350, pH 4.7. The crystals were cryoprotected by Parabar 10312 (previously known as Paratone oil, Hampton Research, USA). X-ray diffraction data were collected on beamline BL18U1 at the Shanghai Synchrotron Radiation Facility at 100 K and at a wavelength of 0.97852 Å. Data integration and scaling were performed using HKL3000^50^. The structure was determined by SeMet single-wavelength anomalous dispersion (SAD) method with the AutoSol program in PHENIX^51^. The CidA^*w*Mel^ model was initially built by the Autobuild program in PHENIX and subsequently subjected to iterative cycles of manual building in Coot^52^ and refinement in PHENIX.

The crystals of the CidA^*w*Pip(Tunis)^-CidB^*w*Pip(Tunis)^_ND1-ND2_ complex were grown at 16 °C from a mixture of 1 µL protein (5 mg/mL) and 1 µL crystallization buffer containing 0.1 M BICINE, pH 8.5 and 15% w/v Polyethylene glycol 1500. The crystals were cryoprotected by Parabar 10312. X-ray diffraction data were collected on beamline BL18U1 at the Shanghai Synchrotron Radiation Facility. The structure was determined by molecular replacement using the structure of the CidA^*w*Pip(Pel)^-CidB^*w*Pip(Pel)^_ND1-ND2_ complex as the search model^53^.

The crystals of the CidA^*w*Mel^(ST)-CidB^*w*Pip(Pel)^_ND1-ND2_ complex were grown at 16 °C from a mixture of 1 µL protein (5 mg/mL) and 1 µL crystallization buffer containing 5% v/v (+/-)-2-Methyl-2,4-pentanediol, 0.1 M HEPES pH 7.5, 10% w/v Polyethylene glycol 10,000. The crystals were cryoprotected by Parabar 10312. X-ray diffraction data were collected on beamline BL18U1 at the Shanghai Synchrotron Radiation Facility. The structure was determined by molecular replacement using the structure of the CidA^*w*Pip(Tunis)^-CidB^*w*Pip(Tunis)^_ND1-ND2_ complex as the search model.

CidB^*w*Mel^_DUB_ was crystallized by hanging drop method at 18 °C, with the crystallization reservoir solution containing 10 mM Nickel (II) Chloride hexahydrate, 100 mM TRIS pH 8.5 and 20% w/v Polyethylene Glycol Monomethyl Ether 2000. The crystals were directly flash frozen in liquid nitrogen using reservoir solution supplemented with 10% glycerol as cryoprotectant. X-ray diffraction data were collected at beamline BL17U1 at the Shanghai Synchrotron Radiation Facility. The structure was determined by SeMet SAD method as described above.

Data collection and structure refinement statistics are summarized in Supplementary Table 1. All Molecular graphics were created using UCSF ChimeraX^54^.

### In vitro pull-down experiments

*E. coli* BL21(DE3) expressing either His-tagged CidA mutants or GST-tagged CidB_ND1-ND2_ variants were harvested by centrifugation and resuspended separately in a buffer containing 20 mM Tris-HCl, pH 8.0, 300 mM NaCl, 1 mM PMSF and 5% glycerol. The expression levels of CidA variants were approximated by the BCA Protein Assay (Tiangen Biotech (Beijing) Co., Ltd., China). Equal amount of CidA variants were mixed with a large and fixed volume of CidB_ND1-ND2_ variants to ensure that CidB_ND1-ND2_ variants were in excess to CidA. The mixture was co-lysed by sonication. Cleared lysates were incubated with 50 μL Ni-NTA resin at 4 °C for 1 h. The resin was washed eight times with 800 μL buffer containing 20 mM Tris-HCl pH 8.0, 200 mM NaCl, 0.01% Tween-20, 50 mM imidazole each time. Proteins were eluted by adding 3 resin volumes of 20 mM Tris-HCl pH 8.0, 200 mM NaCl, 300 mM imidazole. The samples were analyzed using the SDS-PAGE and Coomassie stain.

### Yeast growth assays

Growth was analyzed in the BY4741 strain as previously described^55^. Briefly, yeast cultures were grown overnight at 30 °C in Yeast Extract Peptone Dextrose (YPD) media or synthetically defined (SD) raffinose media lacking uracil, leucine or both. Yeast were pelleted by centrifugation, washed with sterile water, and spotted in six-fold serial dilution from an initial OD_600_ 0.2 concentration on solid minimal synthetic media containing either 2% galactose or glucose and lacking either uracil, leucine, or both. Plates were placed at 30 or 36 °C for 2 to 3 days.

### Western blot analysis

For immunoblotting, co-expression culture in raffinose minimal medium (SD) lacking uracil and leucine were diluted to 0.2 OD_600_ in galactose (inducing) minimal medium (SD) lacking uracil and leucine, kept 12–16 h at 30 °C until reaching 0.8-1.0 OD_600_ at which point the equivalent of 2.5 OD_600_ units of cells were harvested, washed and resuspended in 1 mL dH_2_O followed by the addition of 200 μL dH_2_O and 200 μL 0.2 M NaOH, incubated for 5 min at room temperature. Cells were vortexed intermittently for 20 s and pelleted at 10,000 x g, 1 min. Pellets were stored at −80 °C for at least 15 min, resuspended in 100 μL 1 x SDS-PAGE sample buffer and 4% β-mercaptoethanol then heated at 95 °C for 3 min, centrifuged and 20 μL supernatant were loaded in the 10% SDS-PAGE gel and transferred to PVDF Immobilon-P transfer membranes (0.45 μM pore size) (Sigma-Aldrich) under 70 V, 2.5 h used for immunoblot analyses. Antibodies used for immunoblotting were as following: mouse anti-FLAG M2 (Sigma, 1:10,000), secondary antibody used was sheep anti-mouse NXA931V (GE Healthcare, 1:5,000); and mouse anti-PGK1 (yeast phosphoglycerate kinase; Abcam, 1:10,000), secondary antibody used was sheep anti-mouse NXA931V (GE Healthcare, 1:10,000). All immunoblot analyses used 5% milk for blocking. All serial dilution and Western blot data are representative of at least two biological experiments. Proteins were visualized by HRP-based chemiluminescence^56^.

### AlphaFold modeling

In this study, AlphaFold was used to predict the monomer structure of CidB^*w*Mel^_ND1-ND2_, and AlphaFold-Multimer was used to predict the binding complex of CidA^*w*Mel^-CidB^*w*Mel^_ND1-ND2_ with multiple sequence alignments (MSA) set as the all genetics database used at CASP14. The prediction of complexes was run twice with different random seeds and 10 models were obtained. Beginning with visual inspection, four of them were selected to perform protein structural quality check for the side chain conformations using prime module of Schrödinger2021-3. Eventually, the one complex with the highest quality score was selected for further optimization with subsequent MD simulations.

### Molecular dynamics (MD) simulations

MD simulations were performed by using Desmond package of Schrödinger2021-3^57^ using the OPLS4^58^ force field. The binding model of CidA^*w*Mel^-CidB^*w*Mel^_ND1-ND2_ obtained in the last step was explicitly solvated with TIP3P^59^ water molecules under cubic periodic boundary conditions for a 15 Å buffer region. The overlapping water molecules are deleted and 0.15 M KCl is added, and the systems were neutralized by adding K^+^ as counter ions. Brownian motion simulation was used to relax these systems into local energy minimum states separately. An ensemble (NPT) was then applied to maintain the constant temperature (300 K) and pressure (1.01325 bar) of the systems, and the simulations were started with different random initial velocities. The results were visually analyzed by using Maestro graphical interfaces and the RMSD was calculated based on C-alpha atoms. Produced trajectories were clustered using the Desmond trajectory cluster analysis panel. Finally, the most energetically stable binding complex from the largest cluster of conformation in MD trajectory was selected and minimized again with backbone constraints using the prime module of Schrödinger2021-3. The Ramachandran plot of the eventual model was generated with Schrödinger2021-3.

### Evolutionary conservation analysis

The conservation score per amino acid of CidA and CidB was calculated using the ConSurf server (https://consurf.tau.ac.il/) based on their homologous sequences (specifically, CidA homologs from WOPip1, WOBol1-b, WOPipJHB, WOHa1, WOSol, WORecB, WOMelB, WOSuziB, and WORiB and CidB homologs from WOPip1, WOBol1-b, WOHa1, WOSol, WOMelB, WOMelPop, and WOSuziB). The structures of CidA^*w*Pip(Tunis)^ and CidB^*w*Pip(Tunis)^_ND1-ND2_ served as templates. “Maximun likelihood” and “best model” were selected as the calculation method and the evolutionary substitution model, respectively, for this analysis.

### Reporting summary

Further information on research design is available in the Nature Research Reporting Summary linked to this article.

## Supplementary information

Supplementary Information

Reporting Summary

## Data Availability

The data that support this study are available from the corresponding authors upon reasonable request. The coordinates for crystal structures of CidA^*w*Mel^, CidB^*w*Mel^_DUB_, CidA^*w*Pip(Tunis)^-CidB^*w*Pip(Tunis)^_ND1-ND2_ complex, and CidA^*w*Mel^(ST)-CidB^*w*Pip(Pel)^_ND1-ND2_ complex have been deposited in the Protein Data Bank (PDB), with the accession codes 7FIT, 7FIU, 7FIV, and 7FIW, respectively. Source data are provided with this paper.

## Source data

Source Data
