# Supplementary material for: Crystal Structures of Wolbachia CidA and CidB Reveal Determinants of Bacteria-induced Cytoplasmic Incompatibility and Rescue
Source: Nat Commun. 2022 Mar 25;13:1608. doi: 10.1038/s41467-022-29273-w (PMC8956670; doi:10.1038/s41467-022-29273-w)
Supplement: Supplementary file 1 — Supplementary Information [file 41467_2022_29273_MOESM1_ESM.pdf]

Supplementary Information For

Crystal Structures of *Wolbachia* CidA and CidB Reveal Determinants of Bacteria-induced Cytoplasmic Incompatibility and Rescue

Haofeng Wang[1,2,3,12], Yunjie Xiao[1,2,3,4,12], Xia Chen[1,12], Mengwen Zhang[5,6], Guangxin Sun[5], Feng Wang[1], Lin Wang[2,3], Hanxiao Zhang[1], Xiaoyu Zhang[1,7,8], Xin Yang[4], Wenling Li[1], Yi Wei[1], Deqiang Yao[9], Bing Zhang[2], Jun Li[2], Wen Cui[1,4], Fenghua Wang[1], Cheng Chen[1], Wei Shen[2], Dan Su[10], Fang Bai[2,3], Jinhai Huang[1], Sheng Ye[1], Lei Zhang[1], Xiaoyun Ji[11], Wei Wang[4,*], Zefang Wang[1,8,*], Mark Hochstrasser[5,*], Haitao Yang[1,2,3,8,*]

*Corresponding authors

Email: wangwei@cqmu.edu.cn (W.W.); zefangwang@tju.edu.cn (Z.W.); mark.hochstrasser@yale.edu (M.H.); yanght@shanghaitech.edu.cn (H.Y.);

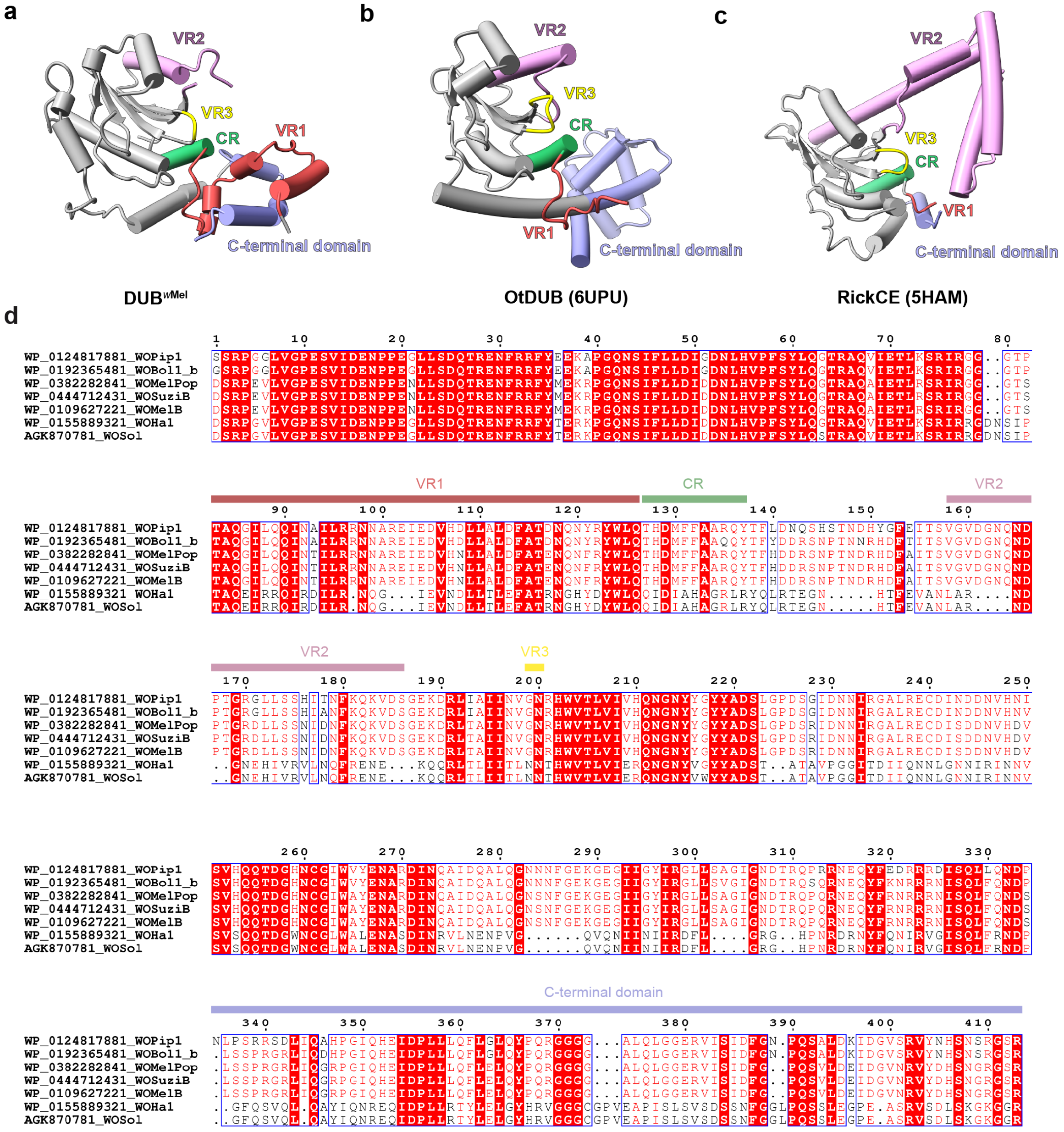

Supplementary Figure 1. The three dimensional structure and phylogenetic analysis of CidB$_{DUB}$. (a) The DUB core (grey) consists of a five-stranded β sheet flanked by α helices on both sides. Based on comparison to other bacterial CE clan deubiquitylases, three variable regions (VR1-VR3), a constant region (CR) and a C-terminal accessory domain may account for the S1 ubiquitin-binding interface. The structures of the OtDUB deubiquitylase from *Orientia tsutsugamushi* **(b)** and RickCE from *Rickettsia bellii* **(c)** are shown for comparison. **(d)** Amino acid sequence alignment of CidB$_{DUB}$ from different *Wolbachia* strains. Conserved residues are shaded. The amino acids at the three VRs, the CR and the C-terminal accessory domain are indicated with solid bars above the alignment.

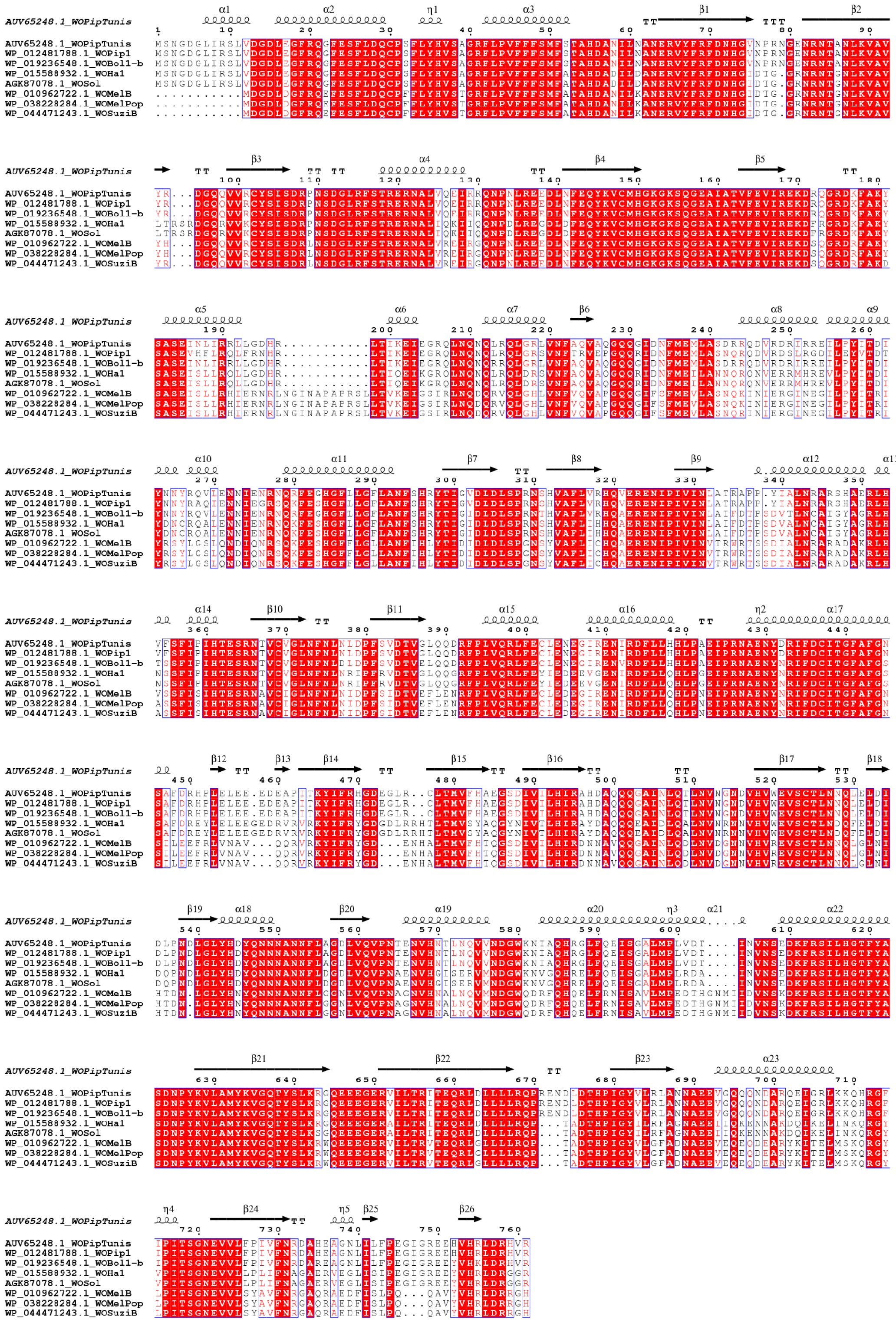

Supplementary Figure 2. Amino acid sequence alignment of CidB$_{ND1\text{-}ND2}$ from different *Wolbachia* strains. Conserved residues are shaded.

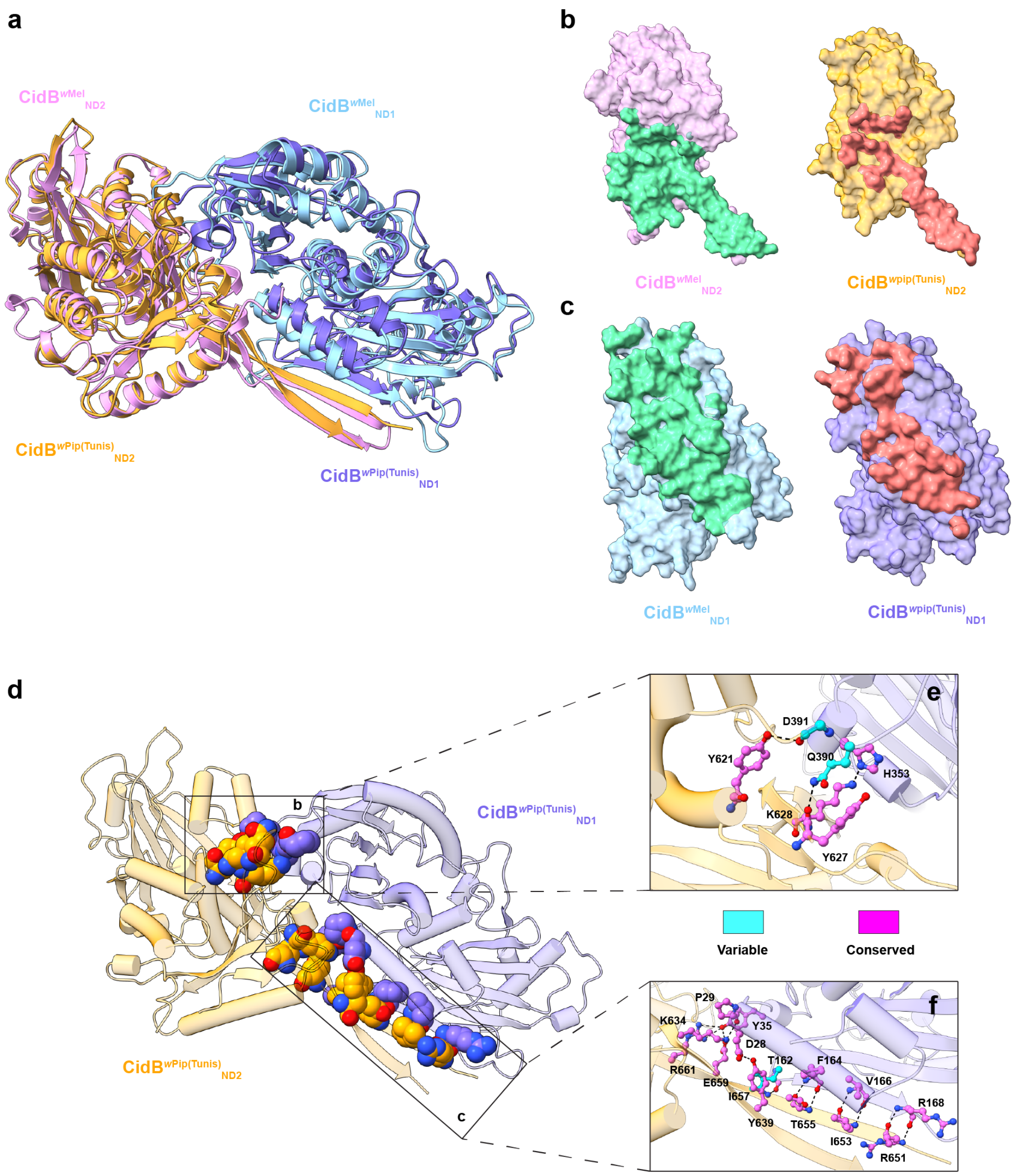

Supplementary Figure 3. The interface between ND1 and ND2 is conserved among CidB homologs. (a) Superposition of the structure of CidB$^{w\text{Pip(tunis)}}_{\text{ND1-ND2}}$ and the model of CidB$^{w\text{Mel}}_{\text{ND1-ND2}}$ shows an identical overall fold and ND1-ND2 interface. **(b, c)** The surfaces involving in ND1-ND2 interaction in each ND domain are highlighted. **(d)** Key residues at the ND1-ND2 interface of CidB$^{w\text{Pip(Tunis)}}$ are shown, which forms a network of hydrogen bond. **(e, f)** The residues which are identical among CidB homologs (from WOPip1, WOBol1-b, WOHa1, WOSol, WOMelB, WOMelPop and WOSuziB; see Supplementary Fig. 2) are in magenta; the residues which are varied are in cyan.

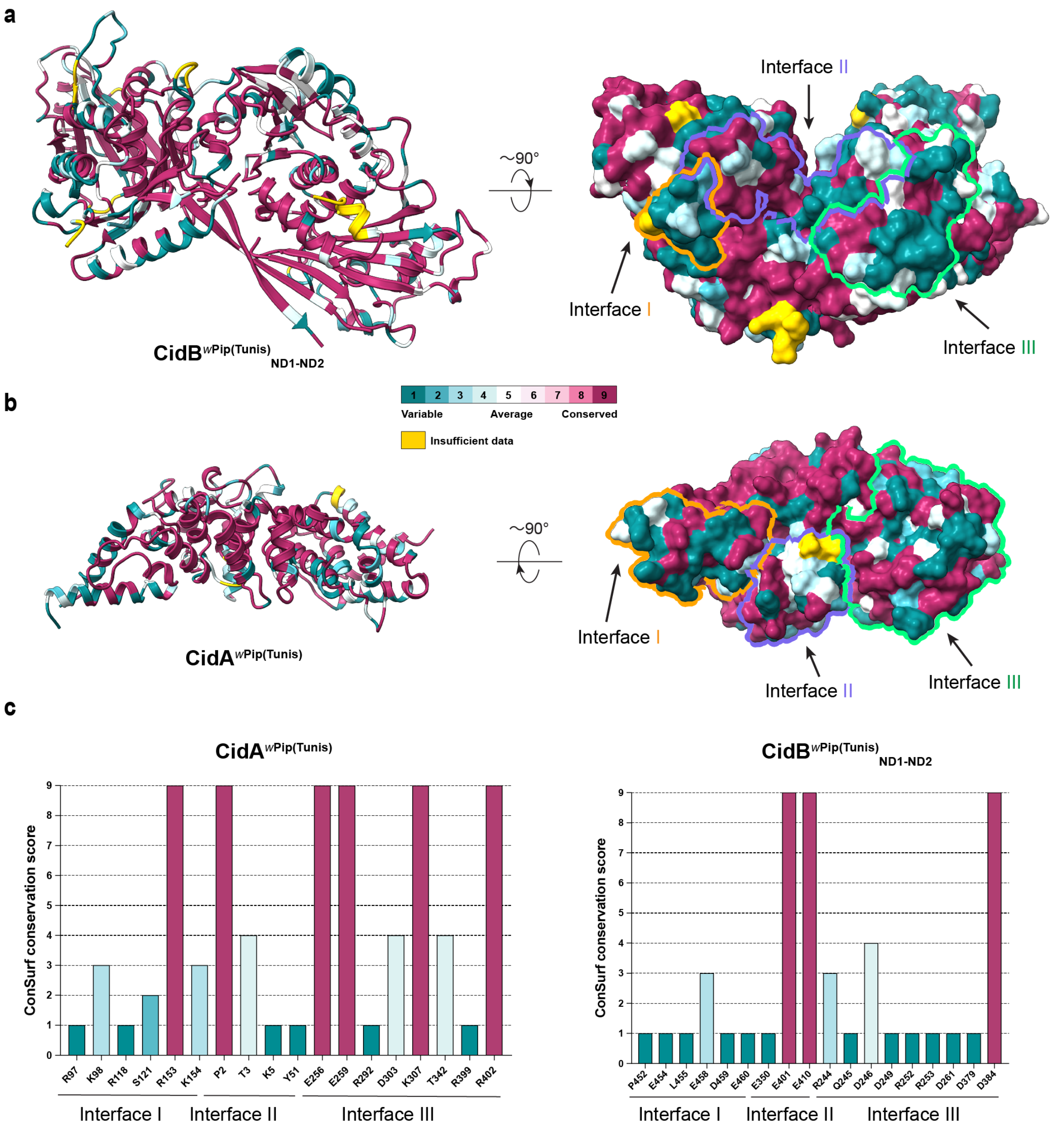

Supplementary Figure 4. The residues at CidA and CidB surface are less conserved than the ones at the center. (a, b) The CidB$^{wPip(Tunis)}$ and CidA$^{wPip(Tunis)}_{ND1-ND2}$ structures in cartoon (left) or surface (right) are colored by the conservation scores calculated by the ConSurf server[1, 2, 3] using sequences of CidA and CidB homologs (specifically, CidA homologs from WOPip1, WOBol1-b, WOPipJHB, WOHa1, WOSol, WORecB, WOMelB, WOSuziB and WORiB and CidB homologs from WOPip1, WOBol1-b, WOHa1, WOSol, WOMelB, WOMelPop and WOSuziB; see Supplementary Fig. 2). The score is a relative measurement of the degree of conservation at each residue position. The conserved positions have a higher

score; whereas the variable positions have a lower score. Insufficient data means that the corresponding positions do not have enough sequences aligned to generate a conservation score. **(c)** The conservation score of key residues contributing to CidA-CidB interaction (see Fig. 2) is shown by histograms.

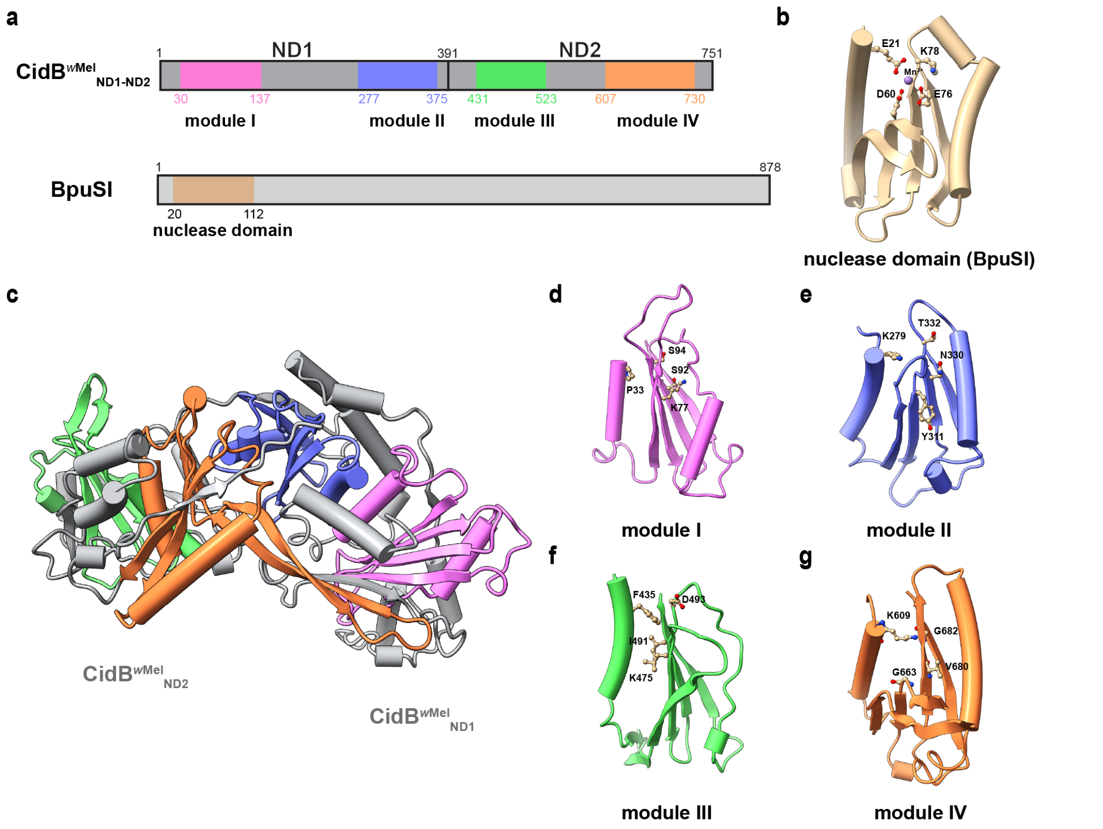

Supplementary Figure 5. Detailed examination on CidB$^{wMel}_{ND1\text{-}ND2}$ structure reveals four α/β modules. **(a)** Schematic diagrams of CidA^{wMel}, CidB$^{wMel}_{ND1\text{-}ND2}$ and BpuSI, a well-characterized nuclease. **(b)** BpuSI has a canonical PD-(D/E)XK nuclease fold (PDB: 3S1S). The catalytic residues are shown as sticks. **(c-g)** CidB$^{wMel}_{ND1\text{-}ND2}$ comprises four α/β modules of the PD-(D/E)XK fold, which are shown in pink (I), blueberry(II), green (III) and orange (IV). The previously predicted PD-(D/E)XK nuclease domains corresponding to modules II and IV. The residues which correspond to the canonical catalytic residues are labeled.

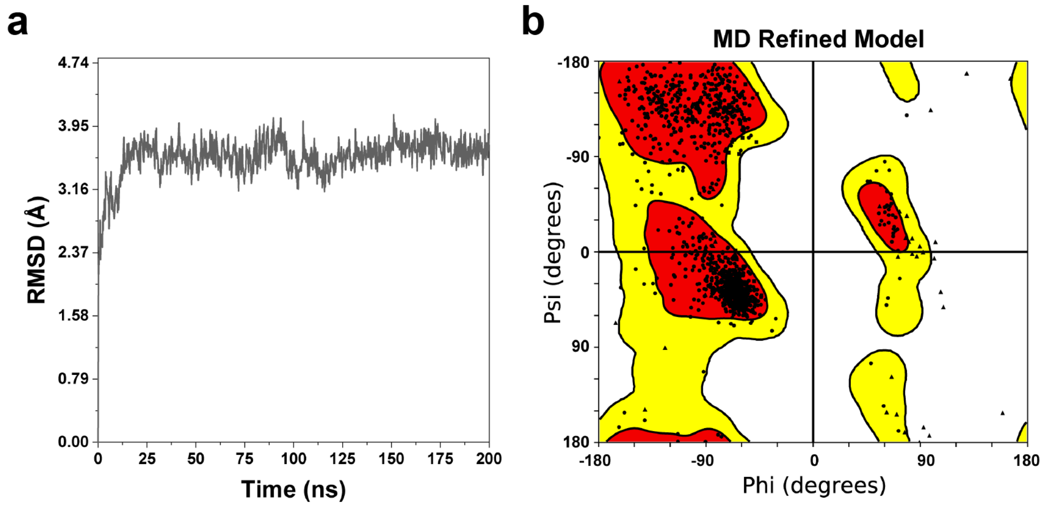

Supplementary Figure 6. Molecular dynamic simulation and validation of the model of the CidA^{wMel}-CidB$^{wMel}_{ND1\text{-}ND2}$ complex. (a) The RMSD of the CidA^{wMel}-CidB$^{wMel}_{ND1\text{-}ND2}$ complex along the simulation trajectory. **(b)** The Ramachandran plot of the optimized CidA^{wMel}-CidB$^{wMel}_{ND1\text{-}ND2}$ binding complex.

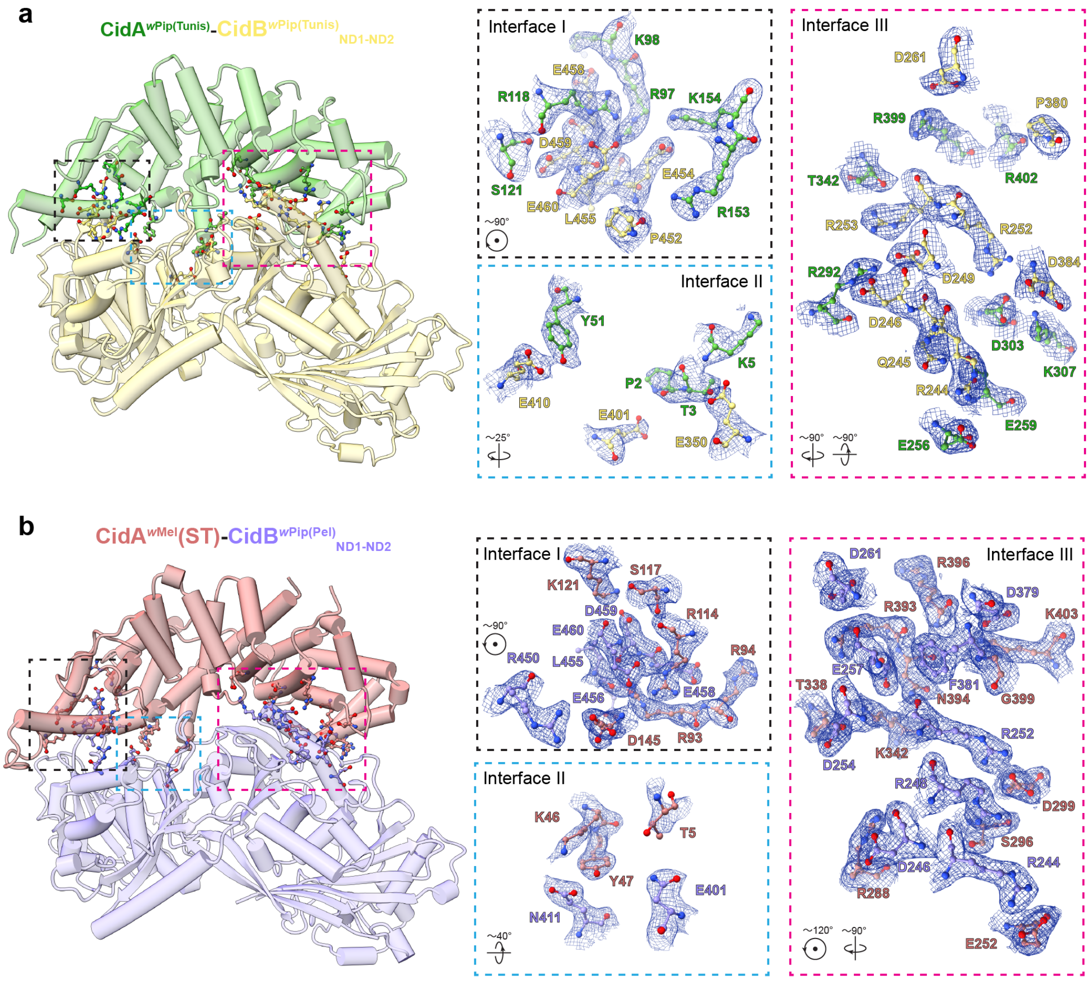

Supplementary Figure 7. Representative electron density for structures of CidA$^{wPip(Tunis)}$-CidB$^{wPip(Tunis)}$$_{ND1-ND2}$ and CidA^{wMel}(ST)-CidB$^{wPip(Pel)}$$_{ND1-ND2}$ complex. (a) Electron density map (2Fo-Fc) around key residues at the interface I, II, III of the CidA$^{wPip(Tunis)}$-CidB$^{wPip(Tunis)}$$_{ND1-ND2}$ complex, contoured at 1.0 σ. **(b)** Electron density map (2Fo-Fc) around key residues at the interface I, II, III of CidA^{wMel}(ST)-CidB$^{wPip(Pel)}$$_{ND1-ND2}$ complex, contoured at 1.0 σ.

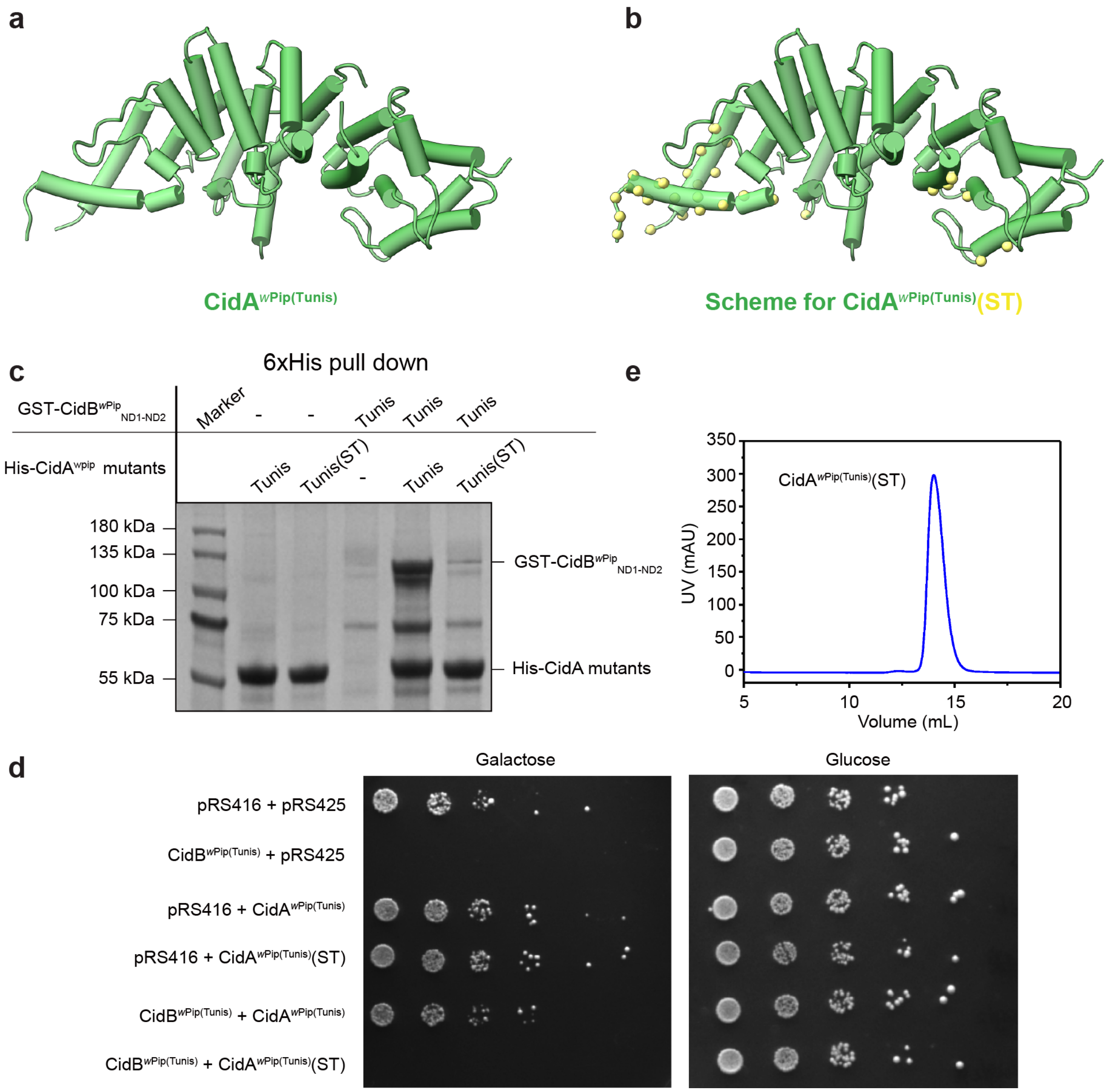

Supplementary Figure 8. Mutagenesis with binding and yeast growth assays reveal the importance of interfacial residues. (a) The structure of CidA$^{w\text{Pip(Tunis)}}$ from the CidA$^{w\text{Pip(Tunis)}}$-CidB$^{w\text{Pip(Tunis)}}{}_{\text{ND1-ND2}}$ complex. **(b)** A chimera named CidA$^{w\text{Pip(Tunis)}}$(ST) with the body of CidA$^{w\text{Pip(Tunis)}}$ (green) and interfacial residues from CidA$^{w\text{Mel}}$. The locations of the mutated residues are shown in yellow on the CidA$^{w\text{Pip(Tunis)}}$ structure. CidA$^{w\text{Pip(Tunis)}}$(ST) cannot **(c)** bind to CidB$^{w\text{Pip(Tunis)}}$ and **(d)** rescue CidB$^{w\text{Pip(Tunis)}}$-induced yeast growth defect. These experiments were repeated three times independently with similar results obtained. **(e)** CidA$^{w\text{Pip(Tunis)}}$ (ST) folds correctly as shown by size exclusion chromatography. Source data are provided at the end of the Supplementary Information.

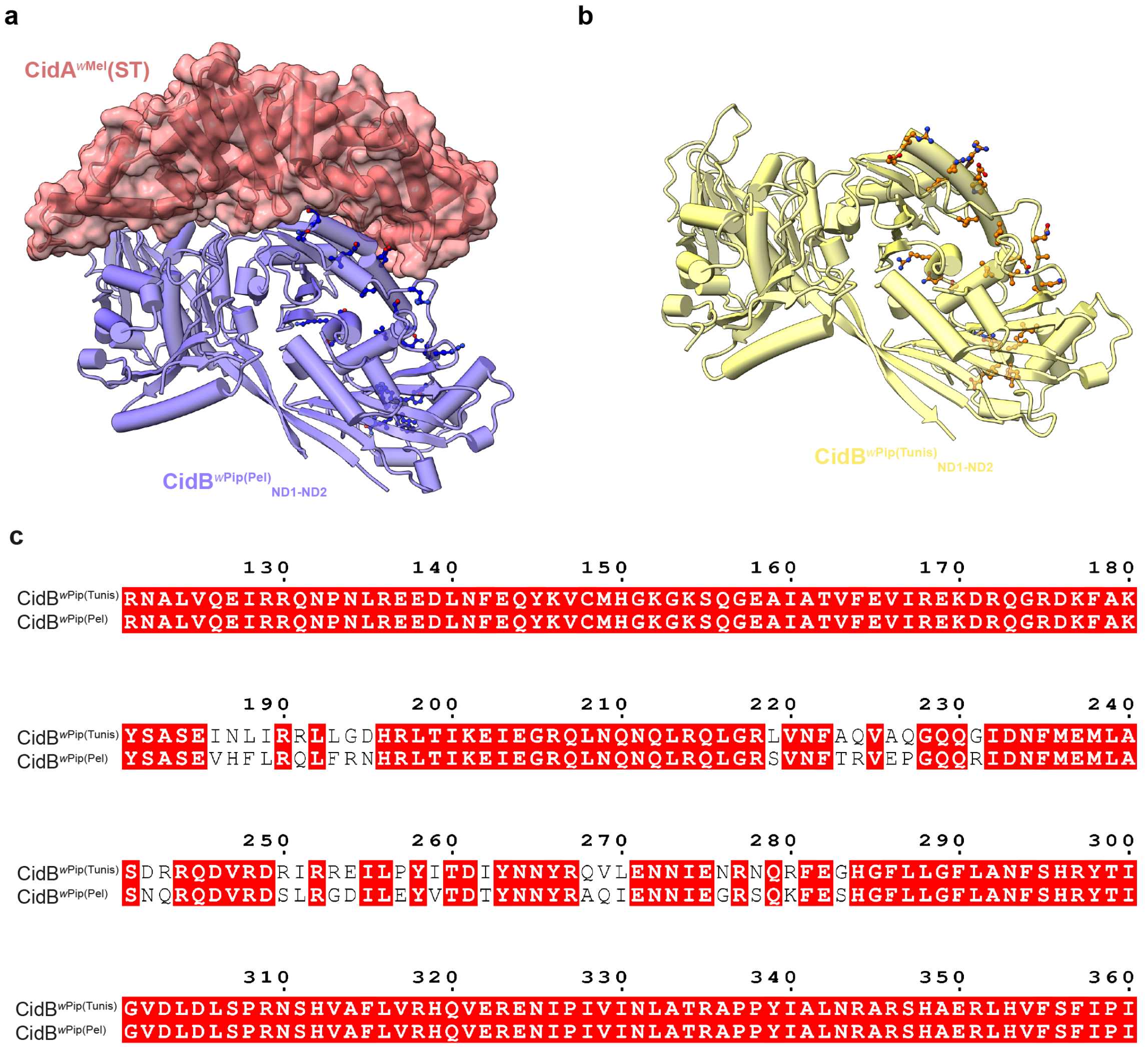

Supplementary Figure 9. The differences between CidB$^{w\text{Pip(Pel)}}$ and CidB$^{w\text{Pip(Tunis)}}$. (a) The crystal structure of the CidA$^{w\text{Mel}}$(ST)-CidB$^{w\text{Pip(Pel)}}_{\text{ND1-ND2}}$ complex. The residues of CidB$^{w\text{Pip(Pel)}}_{\text{ND1-ND2}}$ which are different from CidB$^{w\text{Pip(Tunis)}}_{\text{ND1-ND2}}$ are shown as sticks. The corresponding residues of CidB$^{w\text{Pip(Tunis)}}_{\text{ND1-ND2}}$ are shown as sticks in **(b)**. **(c)** Sequence alignment of CidB$^{w\text{Pip(Pel)}}$ and CidB$^{w\text{Pip(Tunis)}}$. Only the region that has sequence variation is shown.

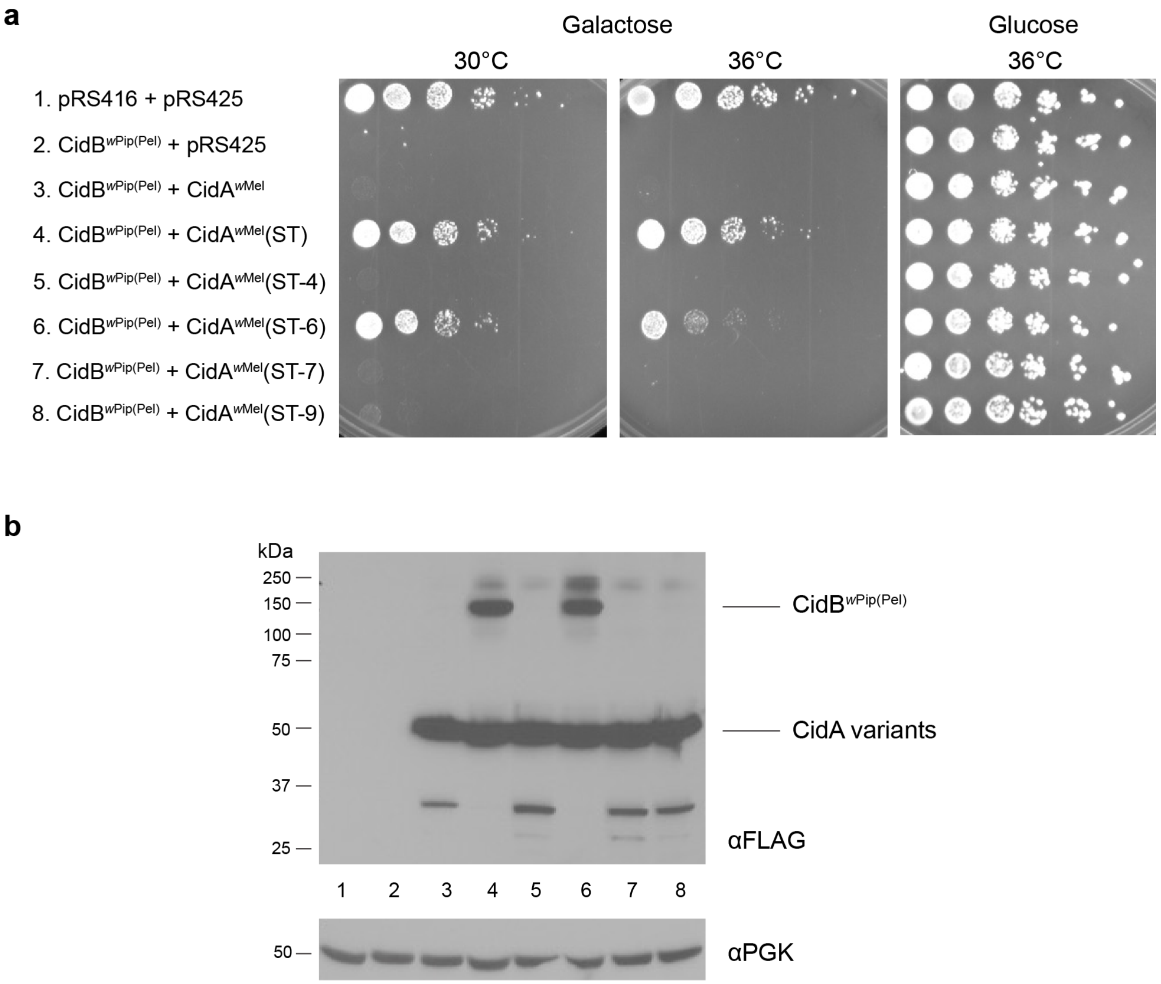

Supplementary Figure 10. CidA^{wMel} can suppress CidB$^{wPip(Pel)}$ toxicity in yeast (BY4741) when its interfacial residues are mutated, as in CidA^{wMel}(ST), to those of the CidA$^{wPip(Pel)}$ antidote protein. **(a)** Genes were expressed from the *GAL1* promoter in the indicated plasmids and a dilution series of each culture was spotted on the indicated plates and incubated for ~2.5 days. **(b)** Immunoblot analysis of the same cultures as in (a). All proteins were N-terminally tagged with a FLAG epitope and migrated at roughly the expected sizes. Similar expression of all the CidA variants was observed. The likely Flag-tagged CidB$^{wPip(Pel)}$ was only detected in the presence of a CidA capable of binding to it (see lanes 4,6), suggesting CidA binding protects CidB from degradation. PGK, loading control. The experiment was repeated three times with similar results obtained. Source data are provided at the end of the Supplementary Information.

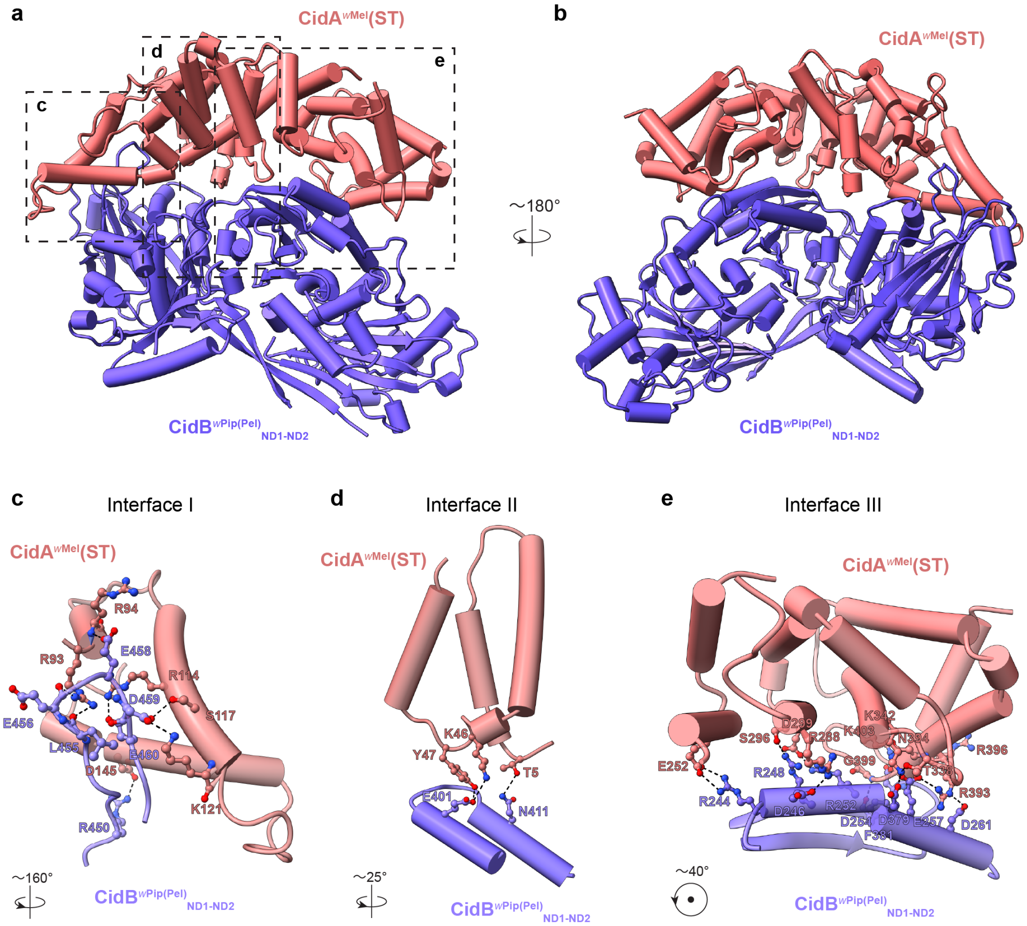

Supplementary Figure 11. CidA^{wMel}(ST) interacts with CidB$^{wPip(Pel)}$ through three interfaces. (a) and (b) The structure of the CidA^{wMel}(ST)-CidB$^{wPip(Pel)}_{ND1-ND2}$ complex. The locations of the three interfaces are labeled. **(c-e)** The details of the three interfaces are shown, with the residues directly involving in the interaction labeled.

Supplementary Table 1. X-ray data collection and refinement statistics

	CidA$^{w\mathrm{Mel}}$	CidA$^{w\mathrm{Pip(Tunis)}}$-CidB$^{w\mathrm{Pip(Tunis)}}{}_{\mathrm{ND1\text{-}ND2}}$	CidB$^{w\mathrm{Mel}}{}_{\mathrm{DUB}}$	CidA$^{w\mathrm{Mel}}$(ST)-CidB$^{w\mathrm{Pip(Pel)}}{}_{\mathrm{ND1\text{-}ND2}}$
**Data collection**				
Space group	*P*63	*P*41	$P2_12_12_1$	$P2_12_12_1$
Cell dimensions				
a, b, c (Å)	109.9, 109.9, 79.1	181.6, 181.6, 54.6	48.11, 60.59, 85.55	82.4, 98.7, 184.8
α, β, γ (°)	90, 90, 120	90, 90, 90	90, 90, 90	90, 90, 90
Wavelength (Å)	0.97852	0.97915	0.97918	0.97915
Resolution limit (Å)	50.00-2.75 (2.85-2.75)*	50.00-2.60 (2.64-2.60)*	50.00-1.85 (1.90-1.85)*	50.00-2.15 (2.19-2.15)*
R_{merge} (%)	16.5 (160.3)	19.0 (131.2)	8.0 (119.9)	17.5 (104.8)
$I / \sigma I$	35.3 (2.6)	12.2 (1.4)	14.3 (1.9)	11.6 (1.3)
Completeness (%)	99.4 (99.0)	100.0 (99.6)	99.2 (98.2)	98.8 (88.0)
Redundancy	17.4 (18.5)	11.1 (6.8)	8.6 (9.2)	10.8 (6.2)
**Refinement**				
Resolution range (Å)	50.00-2.75	50.00-2.60	50.00-1.85	50.00-2.15
No. reflections	14229	56069	22149	80184
R_{work} / R_{free}	0.219/0.257	0.198/0.242	0.210/0.238	0.192/0.220
NO. atoms				
Protein	2862	9242	2127	9433
Ligand/ion	0	0	0	0
Water	29	183	68	643
B-factors				
Protein	92.38	72.23	46.72	49.86
Ligand/ion	/	/	/	/
Water	68.15	56.80	49.48	48.43
**R.m.s deviations**				
Bond lengths (Å)	0.010	0.006	0.005	0.002
Bond Angles (°)	1.06	0.75	0.77	0.52
**Ramachandran statistics (%)**				
Favored	99.43	97.36	98.85	98.42
Allowed	0.57	2.64	1.15	1.49
Outliers	0.00	0.00	0.00	0.09

*Values in parentheses are for highest-resolution shell.

Supplementary Table 2. Primers used to generate CifA or CifB expression plasmids

Construct name	Primer name	Primer sequence (5'-3')
pET-22b(+)-CidA$^{wPip(Tunis)}$	22b-CidA$^{wPip(Tunis)}$-fw	GGAATTCCATATGCCGACCCAGAAAGAACT
	22b-CidA$^{wPip(Tunis)}$-rev	CCGCTCGAGTTTGTTGCCGCTCAGGGTAA
pET-22b(+)-CidB$^{wPip(Tunis)}_{ND1-ND2}$	22b-CidB$^{wPip(Tunis)}_{ND1-ND2}$-fw	GGAATTCCATATGAGCAACGGCGATGGC
	22b-CidB$^{wPip(Tunis)}_{ND1-ND2}$-rev	CCGCTCGAGACGAACGTGACGGTCCAGAC
pET-22b(+)-CidB$^{wPip(Pel)}_{ND1-ND2}$	22b-CidB$^{wPip(Pel)}_{ND1-ND2}$-fw	GGAATTCCATATGTCTAACGGTGACGGTCTGATC
	22b-CidB$^{wPip(Pel)}_{ND1-ND2}$-rev	CCGCTCGAGACGAACGTGACGGTCCAGAC
pET-22b(+)-CidA^{wMel}	22b-CidA^{wMel}-fw	GGAATTCCATATGCCGATCGAAACCAAA
	22b-CidA^{wMel}-rev	CCGCTCGAGGATACGACGCTTCTTGTGAG
pGEX-6p-1-CidB$^{wMel}_{DUB}$	pGEX-6p-1-CidB$^{wMel}_{DUB}$-fw	CTGGGATCCCAGAACTCTATCTTCCTGCTG
	pGEX-6p-1-CidB$^{wMel}_{DUB}$-rev	CCGCTCGAGCGCACCACCACCACCACGCTG
pET28a-GST	pET28a-GST-fw	CATGCCATGGGCATGTCCCCTATACTAGGTTATTGGAAAATTAAGGGCC
	pET28a-GST-rev	CGGGATCCCAGGGGCCCCTGGAACAGAACTT
pET28a-GST-CidB$^{wPip(Tunis)}_{ND1-ND2}$	pET28a-GST-CidB$^{wPip(Tunis)}_{ND1-ND2}$-fw	CGGGATCCATGAGCAACGGCGATGGC
	pET28a-GST-CidB$^{wPip(Tunis)}_{ND1-ND2}$-rev	CCGCTCGAGTTAACGAACGTGACGGTCCAGAC
pET28a-GST-CidB$^{wPip(Pel)}_{ND1-ND2}$	pET28a-GST-CidB$^{wPip(Pel)}_{ND1-ND2}$-fw	CGGGATCCATGTCTAACGGTGACGGTCTGATC
	pET28a-GST-CidB$^{wPip(Pel)}_{ND1-ND2}$-rev	CCGCTCGAGTTAACGAACGTGACGGTCCAGAC
pRS416-CidB$^{wPip(Tunis)}$	pRS416-CidB$^{wPip(Tunis)}$-fw	CGGGATCCATGGACTATAAAGACGATGACGATAAAATGAGCAACGGCGATGGC
	pRS416-CidB$^{wPip(Tunis)}$ -rev	ACGCGTCGACTTAACGAGAACCACGAGAGTTAGAGTGG
pRS416-CidB$^{wPip(Pel)}$	pRS416-CidB$^{wPip(Pel)}$-fw	CGGGATCCATGGACTATAAAGACGATGACGATAAAATGTCTAACGGTGACGGTCTG
	pRS416-CidB$^{wPip(Pel)}$-rev	ACGCGTCGACTTAACGAGAACCACGAGAGTTAGAGT
pRS425-CidA$^{wPip(Pel)}$	pRS425-CidA$^{wPip(Pel)}$ -fw	CGGGATCCATGGACTATAAAGACGATGACGATAAAATGCCGACCCAGAAAGAACT
	pRS425-CidA$^{wPip(Pel)}$ -rev	ACGCGTCGACTTATTTGTTACCAGACAGGGTGAAAAC
pRS425-CidA$^{wPip(Tunis)}$	pRS425-CidA$^{wPip(Tunis)}$ -fw	CGGGATCCATGGACTATAAAGACGATGACGATAAAATGCCGACCCAGAAAGAACT
	pRS425-CidA$^{wPip(Tunis)}$ -rev	ACGCGTCGACTTATTTGTTGCCGCTCAGGGTAA
pRS425-CidA$^{wPip(Tunis)}$(ST)	pRS425-CidA$^{wPip(Tunis)}$(ST) -fw	CGGGATCCATGGACTATAAAGACGATGACGATAAAATGCCAACCCAGCGCGAAC
	pRS425-CidA$^{wPip(Tunis)}$(ST) -rev	ACGCGTCGACTTATTTGTTGCCGCTCAGCGTGAA
pRS425-CidA^{wMel}	pRS425-CidA^{wMel}-fw	CGGGATCCATGGACTATAAAGACGATGACGATAAAATGCCGATCGAAACCAAA
	pRS425-CidA^{wMel}-rev	ACGCGTCGACTTAGATACGACGCTTCTTGTGAG

pRS425-CidA^{wMel}(ST), pRS425-CidA^{wMel}(ST-4), pRS425-CidA^{wMel}(ST-6), pRS425-CidA^{wMel}(ST-7), pRS425-CidA^{wMel}(ST-9)	pRS425-CidA^{wMel}(ST) - fw	CGGGATCCATGGACTATAAAGACGATGACGATAAAATGCCAATCGAAACCAAGAAACAAG
	pRS425-CidA^{wMel}(ST) - rev	ACGCGTCGACTTAGATGCGGCGTTTTTATGGCTG

References

1. Ashkenazy H, et al. ConSurf 2016: an improved methodology to estimate and visualize evolutionary conservation in macromolecules. *Nucleic Acids Res.* **44**, W344-350 (2016).

2. Ashkenazy H, Erez E, Martz E, Pupko T, Ben-Tal N. ConSurf 2010: calculating evolutionary conservation in sequence and structure of proteins and nucleic acids. *Nucleic Acids Res.* **38**, W529-533 (2010).

3. Celniker G, et al. ConSurf: using evolutionary data to raise testable hypotheses about protein function. *Isr. J. Chem.* **53**, 199-206 (2013).

Source data

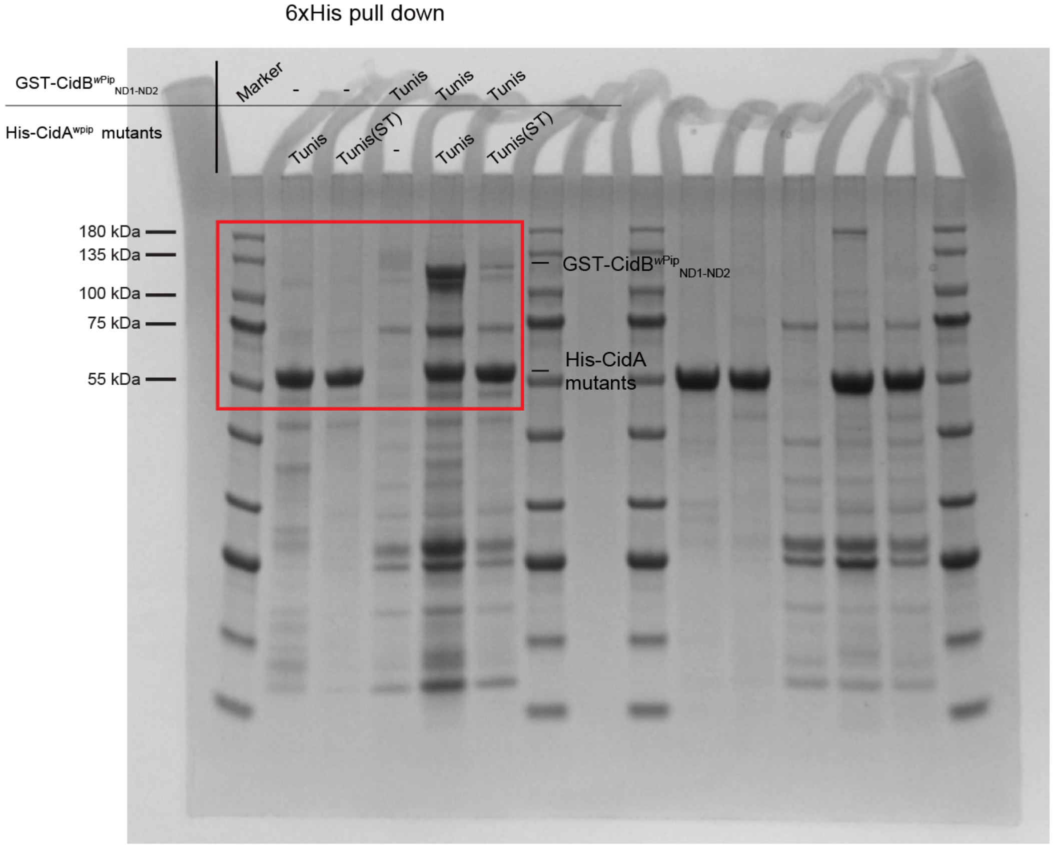

Source data for supplementary Fig. 8c. The part of red box was cropped for Supplementary Fig. 8c as shown.

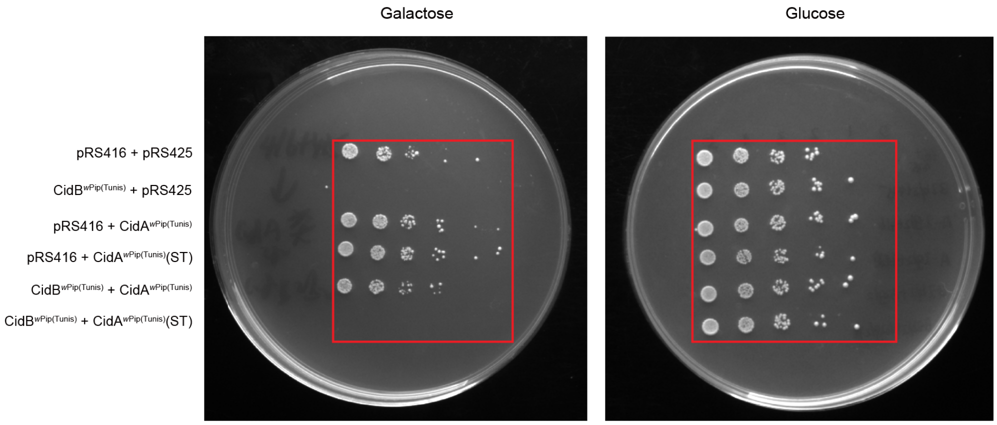

Source data for supplementary Fig. 8d. The part of red box was cropped for Supplementary Fig. 8d as shown.

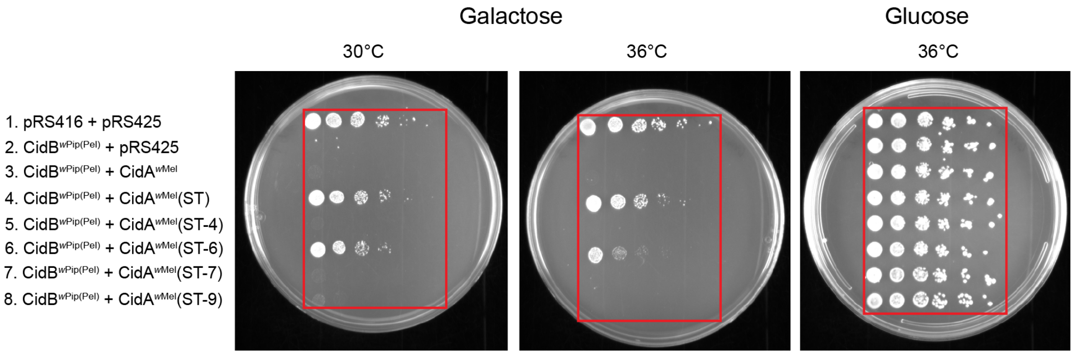

Source data for supplementary Fig. 10a. The part of red box was cropped for Supplementary Fig. 10a as shown.

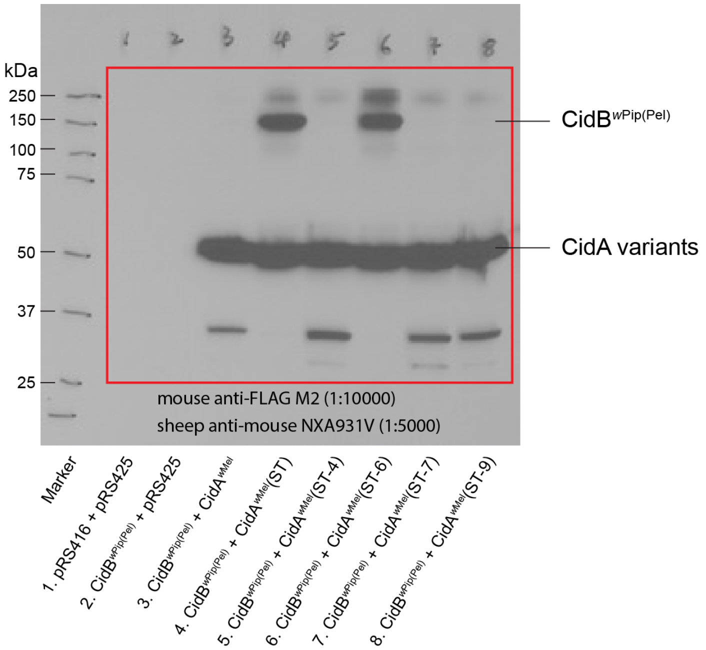

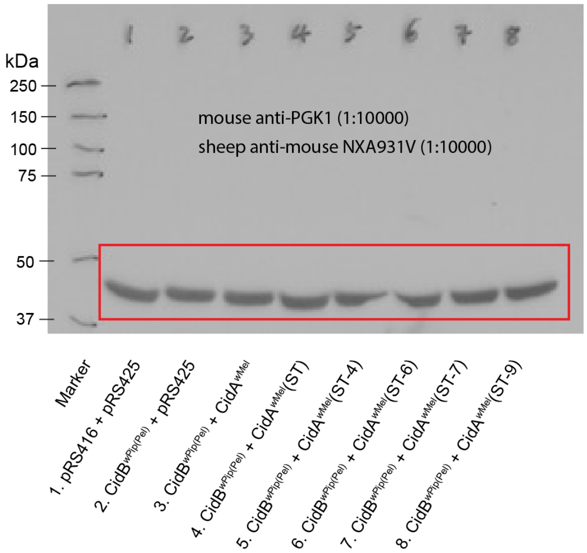

Source data for supplementary Fig. 10b. The part of red box was cropped for Supplementary Fig. 10b as shown.
