# Supplementary material for: Crystal Structures of Wolbachia CidA and CidB Reveal Determinants of Bacteria-induced Cytoplasmic Incompatibility and Rescue
Source: Nat Commun. 2022 Mar 25;13:1608. doi: 10.1038/s41467-022-29273-w (PMC8956670; doi:10.1038/s41467-022-29273-w)
Supplement: Supplementary file 2 — Reporting Summary [file 41467_2022_29273_MOESM2_ESM.pdf]

Corresponding author(s): Wei Wang, Zefang Wang, Mark Hochstrasser, Haitao Yang

Last updated by author(s): February 08, 2022

Reporting Summary

Nature Portfolio wishes to improve the reproducibility of the work that we publish. This form provides structure for consistency and transparency in reporting. For further information on Nature Portfolio policies, see our Editorial Policies and the Editorial Policy Checklist.

Statistics

For all statistical analyses, confirm that the following items are present in the figure legend, table legend, main text, or Methods section.

n/a	Confirmed	
☒	☐	The exact sample size (*n*) for each experimental group/condition, given as a discrete number and unit of measurement
☒	☐	A statement on whether measurements were taken from distinct samples or whether the same sample was measured repeatedly
☒	☐	The statistical test(s) used AND whether they are one- or two-sided<br>*Only common tests should be described solely by name; describe more complex techniques in the Methods section.*
☒	☐	A description of all covariates tested
☒	☐	A description of any assumptions or corrections, such as tests of normality and adjustment for multiple comparisons
☐	☒	A full description of the statistical parameters including central tendency (e.g. means) or other basic estimates (e.g. regression coefficient) AND variation (e.g. standard deviation) or associated estimates of uncertainty (e.g. confidence intervals)
☒	☐	For null hypothesis testing, the test statistic (e.g. *F*, *t*, *r*) with confidence intervals, effect sizes, degrees of freedom and *P* value noted<br>*Give P values as exact values whenever suitable.*
☒	☐	For Bayesian analysis, information on the choice of priors and Markov chain Monte Carlo settings
☒	☐	For hierarchical and complex designs, identification of the appropriate level for tests and full reporting of outcomes
☒	☐	Estimates of effect sizes (e.g. Cohen's *d*, Pearson's *r*), indicating how they were calculated

Our web collection on statistics for biologists contains articles on many of the points above.

Software and code

Policy information about availability of computer code

Data collection	SSRF beamline BL17U1 and BL18U1
Data analysis	HKL3000 721.3, Phenix 1.14, COOT 0.8.9, UCSF Chimera X 1.2.5, Desmond 2021.1, AlphaFold 2.1.1, AlphaFold-Multimer 2.1.1, ConSurf server

For manuscripts utilizing custom algorithms or software that are central to the research but not yet described in published literature, software must be made available to editors and reviewers. We strongly encourage code deposition in a community repository (e.g. GitHub). See the Nature Portfolio guidelines for submitting code & software for further information.

Data

Policy information about availability of data

All manuscripts must include a data availability statement. This statement should provide the following information, where applicable:

- Accession codes, unique identifiers, or web links for publicly available datasets
- A description of any restrictions on data availability
- For clinical datasets or third party data, please ensure that the statement adheres to our policy

The coordinates for crystal structures have been deposited in the Protein Data Bank (PDB), with the accession codes 7FIT (https://doi.org/10.2210/pdb7FIT/pdb), 7FIU (https://doi.org/10.2210/pdb7FIU/pdb), 7FIV (https://doi.org/10.2210/pdb7FIV/pdb) and 7FIW (https://doi.org/10.2210/pdb7FIW/pdb), respectively. Source data are provided with this paper.

Field-specific reporting

Please select the one below that is the best fit for your research. If you are not sure, read the appropriate sections before making your selection.

☒ Life sciences ☐ Behavioural & social sciences ☐ Ecological, evolutionary & environmental sciences

For a reference copy of the document with all sections, see nature.com/documents/nr-reporting-summary-flat.pdf

Life sciences study design

All studies must disclose on these points even when the disclosure is negative.

Sample size	Sample size estimation was not relevant for this study, as it does not report on a statistical evaluation of effects between two or more groups.
Data exclusions	No data were excluded.
Replication	To ensure reproducibility of experimental findings, each assay was performed at least three times to confirm the results. All attempts at replication were successful.
Randomization	Randomization was not required, as all data were performed with recombinant proteins and yeast cells. The identity of the materials used in experiments is known.
Blinding	Animals or human research participants were not involved in this study, so blinding was not required in this study.

Reporting for specific materials, systems and methods

We require information from authors about some types of materials, experimental systems and methods used in many studies. Here, indicate whether each material, system or method listed is relevant to your study. If you are not sure if a list item applies to your research, read the appropriate section before selecting a response.

Materials & experimental systems

n/a	Involved in the study
☐	☒ Antibodies
☒	☐ Eukaryotic cell lines
☒	☐ Palaeontology and archaeology
☒	☐ Animals and other organisms
☒	☐ Human research participants
☒	☐ Clinical data
☒	☐ Dual use research of concern

Methods

n/a	Involved in the study
☒	☐ ChIP-seq
☒	☐ Flow cytometry
☒	☐ MRI-based neuroimaging

Antibodies

Antibodies used	Primary antibodies we used:<br>1) Mouse anti-FLAG M2 (Sigma; catalog# F3165)<br>2) Mouse anti-PGK1 (Abcam; catalog# ab113687)<br>Secondary antibody we used:<br>1) Sheep anti-mouse IgG (GE Healthcare; catalog# NXA931V)
Validation	1) Mouse anti-FLAG M2 (Sigma; catalog# F3165) was validated by Fowler, C. C., Stack, G., Jiao, X., Lara-Tejero, M., & Galán, J. E. (2019). Alternate subunit assembly diversifies the function of a bacterial toxin. Nature communications, 10(1), 3684. Manufacturer's website: https://www.sigmaaldrich.cn/CN/en/product/sigma/f3165.<br>2) Mouse anti-PGK1 (Abcam; catalog# ab113687) was validated by Song, M., Zhai, B., Yang, X., Tan, T., Wang, Y., Yang, X., Tan, Y., Chu, T., Cao, Y., Song, Y., Wang, S., & Zhang, L. (2021). Interplay between Pds5 and Rec8 in regulating chromosome axis length and crossover frequency. Science advances, 7(11), eabe7920. Manufacturer's website: https://www.abcam.com/pgk1-antibody-22c5d8-ab113687.html.
